# Miyake Revisited: Validating the Factor Structure of an Open-Source Cognitive Control Test Battery

**DOI:** 10.5334/joc.480

**Published:** 2026-01-08

**Authors:** Febe Demeyer, Sarah De Pue, Bart Aben, Kirsten A. Verhaegen, Anne-Merel Meijer, Céline R. Gillebert, Eva Van den Bussche

**Affiliations:** 1 Brain & Cognition, KU Leuven, Leuven, Belgium; 2 KU Leuven, Leuven Brain Institute, Leuven, Belgium; 3 HumanTotalCare BV, Department of Research and Development, Utrecht, The Netherlands

**Keywords:** cognitive control, executive control, factor structure

## Abstract

Cognitive control is a prerequisite for achieving goals in daily life. Miyake et al. (2000) distinguished three separable but correlated cognitive control functions in young adults: inhibition, shifting, and updating. This three-factor model was later adapted to a bi-factor model with a common factor and separate updating and shifting factors. Over the years, these models have been replicated in various young adult samples. However, other studies have failed to confirm these models. Furthermore, the variety of tasks used in these studies hampers replication of the underlying factor structure of cognitive control. The primary goal of this study was to address this issue of replicability by validating the factor structure of cognitive control functions using a new test battery based on often-used tasks, while offering full transparency about each step in the analysis process. This test battery comprises nine behavioral tasks measuring inhibition, shifting and updating. The factor structure was assessed in 247 young adults (84.21% female). Confirmatory Factor Analysis was used to test the one-factor model with a common cognitive control function, the three-factor model with separate but correlated cognitive control functions, and the bi-factor model. Our findings supported the three-factor model with correlated cognitive control functions as the best-fitting model, despite some fit indices yielding mixed evidence. Additionally, the test battery in this study is offered as an open-source and easily accessible resource. Finally, we offer a critical look on the field and provide recommendations for future use and adaptations of this test battery to increase its broad applicability.

## Introduction

Cognitive control, also referred to as executive control, is prominent in everyday life and enables us to achieve our goals. We need to exert cognitive control whenever we filter input, select and maintain goal-relevant information, or inhibit irrelevant responses (e.g., Braver et al., 2005; Diamond, 2013). Cognitive control is an umbrella term and comprises a large set of higher-order cognitive functions, such as working memory, attentional control and goal maintenance. Over the years, researchers have developed frameworks to elucidate the complex functional organization of these cognitive control functions and to expose which cognitive and neural processes are involved when we exert cognitive control (e.g., Braver, 2012; Miyake et al., 2000). However, a multitude of definitions and measures of cognitive control are used and different frameworks on the unity and diversity of this construct exist in the literature (Baggetta & Alexander, 2016; von Bastian et al., 2020).

### A Unity and Diversity Framework of Cognitive Control

Early frameworks, such as the multicomponent model of working memory of Baddeley and Hitch (1974) and the Supervisory Attentional System (SAS) of Norman and Shallice (1986), proposed a central executive, responsible for controlling and coordinating all lower-level cognitive processes. Neuropsychological studies attributed these coordinating functions to frontal brain functioning (e.g., Luria, 2012; Rabbitt, 2004; Stuss, 2011), paving the way for a unitary view on cognitive control. As a result, the prefrontal cortex was initially associated primarily with one general cognitive control function (Miller & Cohen, 2001).

However, researchers started to shift away from this unitary view on cognitive control to a more fractional framework of separate but related cognitive control functions. The basis of the unity and diversity framework was first proposed by Teuber (1972), who observed both unity and diversity in patients with prefrontal cortex damage. Depending on the specific location of the prefrontal lesions, impairments were differently expressed in different patients, indicating diversity. However, the impairments in these patients were all related to goal-directed behavior, suggesting a commonality across cognitive control functions (e.g., Stuss & Alexander, 2007). This combination of diversity and unity is also reflected in commonly used cognitive control tasks (Friedman & Miyake, 2017). Although these tasks are often assumed to tap into the same general underlying function (i.e., cognitive control), they often show rather weak correlations (Willoughby et al., 2014), suggesting that cognitive control functions may be more diverse than the unitary view on cognitive control suggested. This unity and diversity framework became widely known by the seminal work of Miyake et al. (2000).

Miyake and colleagues (2000) distinguished three often studied cognitive control functions, namely inhibition, shifting, and updating (Friedman et al., 2008). Inhibition involves the suppression of irrelevant information or unwanted automatic responses to achieve a goal. Shifting or task switching is the process of flexibly switching between different tasks or mental sets. Updating involves storing new information in working memory, monitoring this information and updating it when the context changes and information is no longer relevant. In the study by Miyake and colleagues, a cognitive control test battery with tasks assessing inhibition, shifting and updating was completed by a sample of 137 young healthy adults. Using Confirmatory Factor Analysis (CFA), Miyake et al. (2000) tested to what extent the different cognitive control functions are distinct and to what extent they are related to each other. CFA involves constructing an a priori theoretical factor model and then testing the fit of the model to the observed data (Kline, 1998). Comparing different factor models can provide insight into the functional organization of cognitive control functions.

This approach allowed for addressing the problem of task impurity in cognitive control tasks. This refers to the idea that cognitive control tasks also involve low-level processes (e.g., color processing in the Stroop task) that are not related to cognitive control (Friedman & Miyake, 2017; Miyake & Friedman, 2012). The substantial presence of these processes, in addition to random measurement error, may influence performance on these tasks, making it difficult to measure the cognitive control function of interest in a pure way. By using multiple tasks to assess each cognitive control function and by using a latent variable approach, the common variance that is shared across the cognitive control tasks (i.e., the common underlying latent factor) can be statistically extracted, resulting in a more reliable measure of the cognitive control functions.

**Figure 1:**
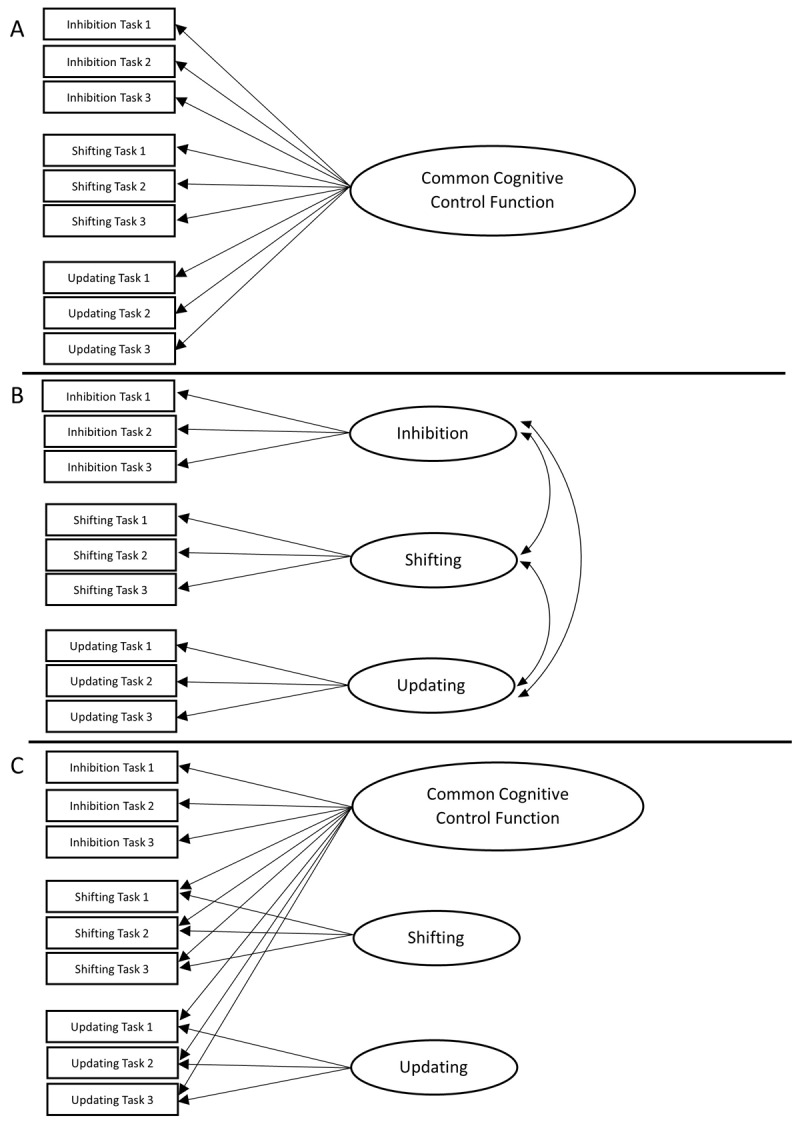
Theoretical latent variable models. *Note*. Panel **A:** One-factor model with a common cognitive control function. Panel **B:** Three-factor model with correlated functions. Panel **C:** Bi-factor model with separate shifting and updating functions. Latent variables are depicted as ellipses and the rectangles represent the dependent measures of the cognitive control tasks that load on these latent variables.

In Miyake et al. (2000), several models with either one, two or three latent variables were fitted. If the three cognitive control functions all tap into the same underlying common cognitive control factor, a one-factor model would have fitted the data best (see Figure 1A). Alternatively, the so-called “full three-factor model”, with inhibition, shifting and updating as three separate but correlated latent factors, should have provided the best fit to the data in case of unity and diversity (see Figure 1B). In addition (not depicted on the figure), smaller, two-factor models were tested to see if two factors (instead of three) tap into the same underlying common cognitive control factor. Finally, if all cognitive control functions are independent of each other and do not tap into a common factor, a three-factor model with no correlations between the functions was expected to provide the best fit to the data. The results showed that the full three-factor model (with correlated factors) yielded the best fit to the observed data. Although these latent variables could be distinguished (i.e., *diversity*), they also showed some *unity*, as reflected in moderate to strong correlations ranging from .42 to .63 between the three cognitive control functions.

### Replications and Further Adaptations to the Framework of Miyake and Colleagues

Following Miyake et al. (2000), several researchers replicated this three-factor model in similar young adult samples (e.g., Friedman et al., 2016; Glisky et al., 2021; Schnitzspahn et al., 2012), but also in children (Lehto et al., 2003; Wu et al., 2011) and older adults (Fisk & Sharp, 2004). In contrast, other developmental studies have provided support for one- or two-factor models, suggesting a dedifferentiation of cognitive control functions in children and older adults and highlighting the unity rather than diversity of cognitive control in these populations (Adrover-Roig et al., 2012; Karr et al., 2018; Lee et al., 2013; van der Ven et al., 2013).

Although the three-factor model of Miyake et al. (2000) has been frequently replicated in young adult samples (e.g., Friedman et al., 2016; Glisky et al., 2021; Schnitzspahn et al., 2012), several other latent factor models have been proposed over time. In addition to the one-factor model and the full three-factor model, models with more than three factors, bi-factor models and nested models also emerged in more recent literature (Garcia-Barrera, 2019; Karr et al., 2018).

Remarkably, several studies failed to find an inhibition-specific factor (e.g., Friedman et al., 2008; Sambol et al., 2023). This has been attributed to the task-impurity problem and to the observation that inhibition tasks, such as the Stroop task or the Stop-Signal task, show low construct validity and poor reliability (Duckworth & Kern, 2011; Hedge et al., 2018). Consequently, different inhibition tasks often show only weak to no correlations with one other (Rey-Mermet et al., 2019, 2020; von Bastian et al., 2020). As observed in Friedman et al. (2008, 2011), the inhibition-specific function correlated almost perfectly with the common underlying cognitive control factor, on which all cognitive control tasks loaded. One interpretation of this absence of a separate inhibition factor could be that the common cognitive control factor is inhibition (Valian, 2015). Inhibition is required in most cognitive control tasks, e.g., during task shifting, you have to focus on the task at hand while inhibiting the other task. An alternative explanation suggests that the common cognitive control factor rather reflects the ability to maintain goals and adapt the processing of incoming information to reach that goal (Friedman et al., 2008; Miyake & Friedman, 2012). Friedman and Miyake (2017) argue that this ability is especially crucial for inhibition tasks, in which distracting information or automatic responses need to be inhibited to reach the goal of the task, and therefore no separate inhibition factor is necessary.

To account for this, Friedman and Miyake (2017) adapted the three-factor model of Miyake et al. (2000) and attempted to capture the unity and diversity of cognitive control in a new model, illustrated in Figure 1C. Their bi-factor model has two separate latent factors reflecting shifting and updating (i.e., diversity), and a common cognitive control factor (i.e., unity), which is assumed to load on all tasks and represents “one’s ability to actively maintain task goals and goal-related information” (Miyake & Friedman, 2012, p. 3). This bi-factor model showed a good fit to the data, equally well as the fit of the full three-factor model, and has been replicated in other studies (e.g., Ito et al., 2015; Karr et al., 2018).

### The Current Study

Although the full three-factor model of Miyake et al. (2000) and the bi-factor model of Friedman and Miyake (2017) have been replicated several times, a review by Karr et al. (2018) and a recent study by Sambol et al. (2023) showed that these models are not always a good fit to the data, or even fail to converge (i.e., convergence of a model is achieved when the specified model successfully reaches a solution during the estimation process and the set of parameter estimates found cannot be further improved; Brown, 2015). One possible explanation for these mixed findings may be that studies so far used a wide variety of cognitive control tasks, making it difficult to replicate findings. Therefore, we aimed to validate the factor structure of this test battery by evaluating and comparing different, commonly studied models (see Figure 1). In addition, we aimed to develop a test battery with three tasks for each cognitive control function, and to make it openly available and easily accessible for widespread use, which would aid its broad applicability and adaptability. This adaptability is intended to make the battery suitable for diverse healthy and clinical populations, including children and older adults. To achieve this, tasks were selected that are commonly used in the literature for the widest possible audience. Importantly, the goal of the present study was not merely to replicate previous work, but to do so with full transparency and openness, reporting each step of the analysis process in detail, openly sharing our data and tasks, and situating the study within ongoing debates in the field. This open science approach invites scrutiny and replication, which can ultimately contribute to both theoretical and methodological advancements.

## Method

This study was approved by our institution’s ethical committee. All data, materials (i.e., the cognitive control test battery) and code are publicly available on the Open Science Framework (OSF; https://osf.io/vtpjb/).

### Participants

A total of 286 participants took part in this study. They were undergraduate students and participated for course credit. Participants could only participate if they had a thorough knowledge of Dutch, normal or corrected-to-normal vision, no colorblindness, no psychiatric illness or neurodegenerative disease, no history of a cancer treatment, no reported drugs or alcohol abuse and no medication use with a known influence on cognitive functioning. Participants under the age of 18 could only participate if they obtained consent of a parent or legal guardian. Two participants were excluded for not having completed one of the tasks. After data cleaning procedures (described below), a final sample of 247 participants was included for data analysis, of whom 39 were male. The mean age was 18 years (*SD* = 0.85, range 17–24). Data on race and ethnicity were not collected. However, a WEIRD (i.e., Western, educated, industrialized, rich and democratic) sample resulted from our sampling method of recruiting undergraduate students who participated in exchange for course credit.

### Material

In addition to the cognitive control test battery, participants completed a reaction time (RT) task, the NASA Task Load Index (Hart & Staveland, 1988), a demographic questionnaire and the Raven Standard Progressive Matrices (RSPM; Raven, 2006) to describe our sample.

#### Cognitive Control Test Battery

The cognitive control test battery, available on OSF, comprised nine computerized tasks, three for each cognitive control function. All computer tasks were programmed in Python using the PsychoPy toolbox (v2021.2.3; Peirce et al., 2019). Stimulus sizes were all represented in visual degrees (except for the Plus-Minus task, which used pixels as unit). The participant’s distance from the screen was 57 cm. Tasks for this test battery were selected based on their broad applicability and adaptability to other populations. All tasks were adapted from Miyake et al. (2000) except two (i.e., Go No-Go and N-Back). The Go No-Go task replaced the Stop-Signal task, and the N-Back task replaced the Tone Monitoring task because these tasks use auditory stimuli, making group administration difficult. This adjustment ensured that all tasks could be administered to multiple participants simultaneously while maintaining a variety of stimuli (i.e., words, letters, images) to maximize engagement. Additionally, these tasks can be adapted for different populations, such as children and older adults, with minor modifications (e.g., adjusting response deadlines). For the current study, the tasks were administered in Dutch, but an English translation of the test battery is provided on OSF. All cognitive control tasks were preceded by verbal instructions given by the experimenter combined with written on-screen instructions. For each task, we took the dependent measure that has been most studied. For the dependent measures of each task, we used either error rates or RTs, so that lower scores indicate better performance (i.e., fewer errors or shorter RTs). Table 1 provides a brief description of each task and the dependent measure for each task. We refer to Supplementary Material 1 for a detailed description of the cognitive control tasks used in this test battery.

**Table 1:** Overview of the Cognitive Control Tasks and the Included Dependent Measures for each Task.


COGNITIVE CONTROL FUNCTION	TASK	TASK DESCRIPTION	DEPENDENT MEASURE

Inhibition	Antisaccade	Participants must inhibit the tendency to make a saccade to a cue in order to detect the briefly presented target stimulus	The proportion of errors across all trials

	Go No-Go	Participants must respond to squares of one color and withhold responses to the other color	The proportion of commission errors (i.e., errors on No-Go trials)

	Stroop	Participants must respond to the ink color of the color words while ignoring the meaning of the color word	Congruency effect: The difference between the median RTs on correct incongruent trials and the median RTs on correct congruent trials

Shifting	Local-Global	Participants must switch between responding to either the global or the local feature of a stimulus	Switch cost: The difference between the median RTs on correct switch trials and the median RTs on correct no-switch trials

	Plus-Minus	Participants need to add 3 to each two-digit number in the first block and subtract 3 in the second block. In the last block, they must switch between adding and subtracting 3	Switch cost: The difference between the time to complete the alternating block and the average time to complete the addition and subtraction blocks

	Number-Letter	Participants must switch between classifying a number as even or odd and a letter as a vowel or consonant. A cue indicates which task the participant needs to perform	Switch cost: The difference between the median RTs on correct switch trials in the mixed block and the average median RTs on correct trials in the no-switch blocks

Updating	Keep Track	Participants must remember every last word presented from each category of a serially presented word stream	The proportion of errors across all words

	Letter Memory	Participants must remember the four last letters of a serially presented letter stream	The proportion of errors across all letters

	N-back	Participants must respond to a picture if it matches the picture 2 trials back	The proportion of omission errors (i.e., errors on Hit trials)


#### NASA Task Load Index

The NASA Task Load Index (Hart & Staveland, 1988) measures the subjective performance and subjective mental effort that participants experience during a task. This questionnaire consisted of two questions, asking participants to estimate how successfully they were able to perform the task and how much effort they had to exert to achieve their performance level. Participants had to complete this questionnaire on paper after completing each cognitive control task. Participants indicated their response on a 21-point Likert scale anchored by bipolar descriptors, ‘perfect’ to ‘failure’ for the Subjective Performance Index and ‘very low’ to ‘very high’ for the Subjective Mental Effort Index. The 21 points on the scale ranged from 0 to 100 in increments of five. In this way, higher scores indicate poorer subjective performance and more mental effort, conversely, lower scores indicate better subjective performance and less mental effort.

#### Demographic Questionnaire

This questionnaire covered some demographic questions such as color blindness, sleep, coffee and alcohol consumption, and medical history.

#### Raven Standard Progressive Matrices

At the end of the experiment, the RSPM (Raven, 2006) was assessed on paper to measure fluid intelligence. Participants were given a booklet of 60 matrices consisting of patterns with a missing piece. Questions were listed in order of increasing difficulty. The participant’s task was to select the missing piece from six to eight choices. The test score of a participant consists of the number of correctly completed questions. Based on this test score and percentile norms, a percentile score was calculated to interpret the results.

### Procedure

Participants were tested in a group setting in university computer rooms, with an average of 9 participants per session (*SD* = 5, range 2–21). Upon arrival, participants were asked to read the information letter and informed consent. Additional safety measures regarding COVID-19 were taken (e.g., wearing face masks, disinfecting keyboards, sanitizing hands, etc). After giving consent, participants started with the cognitive control tasks and completed the NASA Task Load Index after each task. Participants were encouraged to ask questions if anything was unclear. The order in which the nine cognitive control tasks were presented varied between test sessions. In establishing these task orders, we ensured that there was sufficient alternation between longer and shorter tasks and that the successive tasks targeted different cognitive control functions. There were six different orders in which the tasks could be presented and the order of the tasks was the same for each participant in the same session (see Supplementary Table 1, for all orders in which the nine tasks were presented). Participants were instructed to start each task after the verbal instruction for that particular task. Each task started with the participant being asked to enter their participant number, biological sex, age and handedness into the computer. After completing this information, participants could additionally read the written instructions on their computer screens for the current task. After the cognitive control tasks, participants were instructed to complete the simple reaction time task. Finally, participants completed the demographic questionnaire and the RSPM. Regular breaks were provided both within tasks (see Supplementary Material 1 for task-specific details) and between tasks to reduce participant fatigue. Between tasks, short pauses were included while the experimenter waited for all participants to finish the task before giving verbal instructions for the next task. The entire experimental procedure took maximally 3 hours.

### Data Cleaning and Transformations

Practice trials for all tasks and incorrectly solved trials for the tasks with RT-based dependent measures were disregarded and the dependent measures (i.e., RTs or error rates) were calculated. To achieve multivariate normality, these data were then examined and corrected for outliers. We used a roughly similar data cleaning procedure as Miyake et al. (2000). First, between-participant distributions were examined for each task separately. Any participant with an overall mean error rate or overall mean RT across conditions of more than three *SD*s above or below the group mean, on one or more of the tasks, was removed before analysis. This procedure affected 23 participants out of 284 (8.10%) in our sample. In addition, for tasks with RT-based dependent measures, within-participant RT distributions were examined and extreme RTs within each participant were discarded. Any trial with an RT that was more than three *SD*s above or below the overall individual mean RT across conditions on a given task, was removed. This procedure affected on average 2.03% of the trials in the tasks with RT based dependent measures. Note that this within-participant cleaning procedure could not be applied to the Plus-Minus task, as this task only has one trial per block.

After this two-step data cleaning method, multivariate normality was still not achieved. For this reason, the dependent measures were transformed to ensure that these measures were multivariate distributed. For the error-rate based measures, we applied an arcsine transformation similar to Miyake et al. (2000). This transformation creates more dispersion in ceiling and floor effects, with the aim of moving the mean error rates towards more normally distributed values (Judd et al., 2017). For the RT based measures, six different transformations were applied on each RT based measure and the transformation that showed the greatest improvement in terms of univariate normality was selected per task (see Supplementary Table 2 for an overview of the univariate statistics after several transformations). Both the Local-Global task and the Plus-Minus task did not show any improvements in univariate normality by transforming the dependent measure. Therefore, the dependent measures of these two tasks were not transformed. We chose a square root transformation for the Stroop task and a cube root transformation for the Number-Letter task. After these transformations, all dependent measures showed improved univariate skewness and/or kurtosis levels (except for the Local-Global task and the Plus-Minus task, which were not transformed), implying improved univariate normality (see Supplementary Table 3, for an overview of the univariate skewness and kurtosis levels after each data cleaning procedure). However, still not all dependent measures were significantly univariately normally distributed and consequently the multivariate normality assumption was not met.

**Figure 2:**
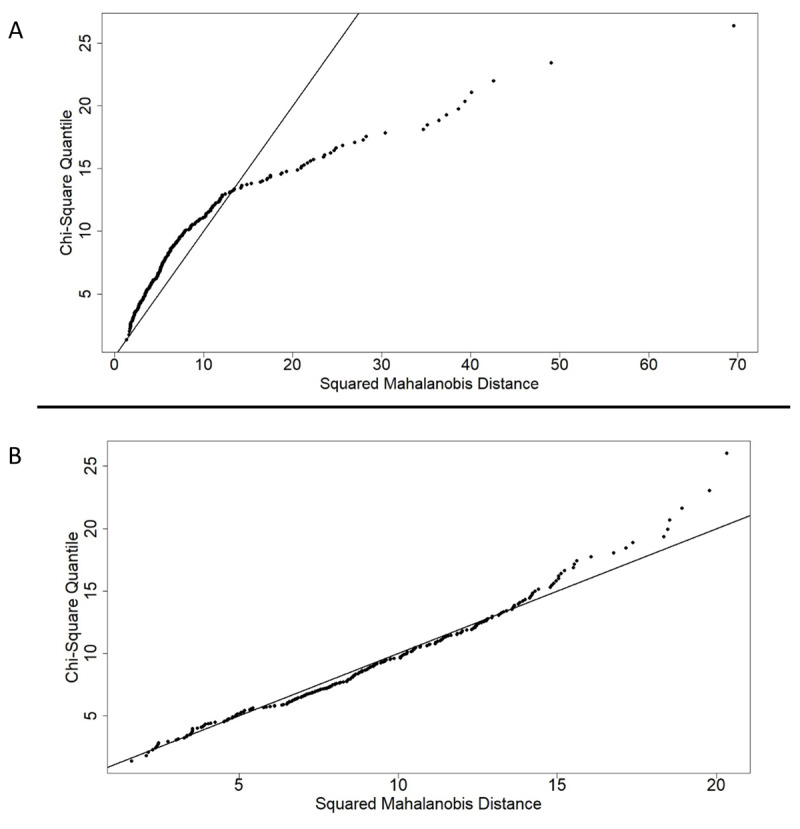
Chi-square Q-Q plots. *Note*. This figure shows the ordered Mahalanobis distances versus the estimated quantiles. Panel **A:** Chi-square Q-Q plot before all data cleaning steps. A clear deviation from the multivariate normal distribution can be observed. Panel **B:** Chi-square Q-Q plot after all data cleaning steps, indicating acceptable multivariate normality.

In a final data cleaning step, we therefore removed 14 multivariate outliers indicated by significant squared Mahalanobis distance values. The squared Mahalanobis distance value is used to find the distance between two points in a multidimensional space and, in this case, indicates the distance between the profile of scores for a participant and the center of the nine-dimensional space (Kline, 1998). In other words, it identifies participants who contribute the most to multivariate non-normality. These participants have outlying scores on more than one task. After removing the multivariate outliers, a sample of 247 participants remained and the multivariate normality assumption was met according to the Mardia’s skewness and kurtosis indices (Korkmaz et al., 2014; Mardia, 1970). These indices assess whether the observed data shows violations of a multivariate normal distribution. The Mardia’s skewness and kurtosis indices were calculated after each data cleaning step and are displayed in Table 2. The Chi-square Q-Q plot indicating acceptable multivariate normality according to the squared Mahalanobis’s distance values, is illustrated in Figure 2B. The Chi-square Q-Q-plot is used to evaluate the agreement between two probability functions (Korkmaz et al., 2014). The X-axis refers to the hypothesized quantiles of the probability distribution and Y-axis refers to the observed quantiles of the probability distribution. The points in the Chi-square Q-Q plot lie on a straight line in case the observed data fits the hypothesized distribution. Deviations from this straight line might indicate that the data is not multivariate normally distributed.

**Table 2:** Normalized Multivariate Skewness and Kurtosis in Each Data Cleaning Step Using Mardia’s Skewness and Mardia’s Kurtosis Indices.


DATASET	MARDIA’S SKEWNESS	MARDIA’S KURTOSIS

Untrimmed data	1495.73 (*p* < .001)	33.42 (*p* < .001)

Participant outliers removed	779.02 (*p* < .001)	16.40 (*p* < .001)

+ Trial outliers removed	756.67 (*p* < .001)	15.23 (*p* < .001)

+ Transformations executed	256.28 (*p* < .001)	6.24 (*p* < .001)

+ Multivariate outliers removed	158.90 (*p* = .619)	–1.64 (*p* = .101)


*Note*. The data were not multivariate normally distributed after each data cleaning step as indicated by significant *p*-values, except for the last step in the cleaning procedure where multivariate outliers were additionally removed.

Since the data cleaning in order to meet the assumption of multivariate normality was substantial, we performed additional analyses on the full, uncleaned dataset (*N* = 284), which only excluded practice trials and incorrectly solved trials for tasks with RT-based dependent measures. The Spearman and Pearson correlation matrices were calculated (see Supplementary Table 2 and Supplementary Table 3, respectively). We conducted CFAs on the uncleaned dataset for all three models (see Supplementary Figures 1–3). We observed that some factor loadings that were significant in the cleaned dataset were no longer significant in the uncleaned dataset. We also computed model fit indices (see Supplementary Table 4). Although the fit indices showed slight changes after data cleaning, the cutoff status of each fit index remained the same (except for the *χ*^2^ test). Taken together, these analyses led to a similar overall interpretation of the results. Therefore, we believe that the data cleaning process did not remove meaningful or consistent covariance among tasks, but rather enhanced the clarity of the underlying structure by reducing noise.

Statistics on the univariate distributions of all nine dependent measures (i.e., skewness, kurtosis and Shapiro-Wilk test values) for the cleaned data as well as the reliability estimates for the dependent measures are provided in Table 3. According to Byrne (2016) and Hair et al. (2010), data are considered to be normal if skewness is between –2 and 2 and kurtosis is between –7 and 7. Given this rule of thumb, all nine dependent measures designed to index the three cognitive control functions showed very good levels of skewness and kurtosis after data cleaning, although not all measures achieved univariate normality according to the Shapiro-Wilk’s test (Shaprio & Wilk, 1965).

**Table 3:** Univariate Statistics for the Outcome Measures of the Nine Cognitive Control Tasks.


TASK	SKEWNESS	KURTOSIS	SHAPIRO-WILK	RELIABILITY

Antisaccade	0.47	0.30	.95	.81

Go No-Go	0.14	0.23	.97	.67

Stroop	0.37	–0.01	.99	.20

Local-Global	–0.08	–0.30	**.99**	.20

Plus-Minus	0.23	0.14	**.99**	

Number-Letter	–0.07	0.23	**>.99**	.87

Keep Track	–0.29	0.29	.98	

Letter Memory	–0.04	–0.25	**.99**	.59

N-back	–0.12	–0.38	.94	.58


*Note*. The univariate statistics are based on the data after cleaning procedures. Measures with a univariate normal distribution according to the Shapiro-Wilk test statistic values are shown in bold (*p* > .05). No reliability estimates could be obtained for the Plus-Minus task and the Keep Track task. The Plus-Minus task only delivers one RT per condition. For the Keep Track task, permutation-based split-half reliability was not possible with only 6 trials per participant. Cronbach’s alpha could also not be computed as the stimuli were randomly presented. For the Antisaccade and Letter Memory tasks, all trials were used to compute split-half reliability. For the Go No-Go and N-back tasks, only trials that were used to compute the dependent measures were used to compute split-half reliability (i.e., No-Go trials for the Go No-Go task and Hit trials for the N-back task). For the Stroop and Local-Global tasks, there were an equal number of trials of each condition in each split (i.e., congruent and incongruent trials for the Stroop task and switch and no-switch trials for the Local-Global task). For the Number-Letter task, the trials in the pure number and letter classification blocks and the switch trials in the mixed block were used to compute split-half reliability. There were an equal number of pure number and letter classification trials and switch trials in each split.

To obtain reliability estimates for each dependent measure, permutation-based split-half reliability was calculated using the splithalf R package (Parsons et al., 2019). The reliability estimates were calculated on the raw data, after removal of the participant, trial and multivariate outliers. The raw data for each task were then randomly split into two parts 10,000 times and the split-half correlation between the dependent measure on each first split and on each second split was calculated. These 10,000 reliability estimates were then averaged and the split-half correlation for each task was adjusted with the Spearman-Brown propensity formula (Brown, 1910; Spearman, 1910). This adjustment accounts for the underestimation caused by halving the total number of trials to calculate a correlation.

### Statistical Analysis

Descriptive statistics were first computed for the NASA Task Load Index, the Reaction Time task, the RSPM and the performance on the nine cognitive control tasks. Pearson’s correlations were then calculated between each two tasks. Correlations were considered weak, moderate or strong if the correlation was less than .30, between .30 and .50 and greater than .50 (Cohen, 1988).

To examine the latent factor structure of cognitive control functions in young adults, we conducted CFAs based on Maximum Likelihood (ML) estimation on the covariance matrix to estimate the latent variable models, using the lavaan package in R (Rosseel, 2012). A one-factor latent model assuming unity of cognitive control (see Figure 1A), a three-factor model assuming both unity and commonality among the three cognitive control functions (see Figure 1B), and a bi-factor model assuming a common cognitive control latent variable and two nested latent variables (i.e., shifting and updating; see Figure 1C) were evaluated. The Antisaccade, Go No-Go and Stroop tasks were used as indicators for the inhibition factor, the Local-Global, Plus-Minus and Number-Letter tasks were indicators for the shifting factor, whereas the Keep Track, Letter Memory and N-back were included as indicators for the updating factor. The variances of all latent variables in all three CFA models were set to one in order to allow free estimation of all factor loadings. The best-fitting model was then selected based on model fit and the strength and direction of the relations between the tasks and the latent variables.

Model fit was assessed using a variety of fit indices (Akaike, 1973; Browne & Cudeck, 1992; Hu & Bentler, 1999): The *χ*^2^ test, the Comparative Fit Index (CFI), the Goodness of Fit Index (GFI), the Adjusted Goodness of Fit Index (AGFI), the Root Mean Square Error of Approximation (RMSEA), the Standardized Root Mean Square Residual (SRMR). The *χ*^2^ test measures and the overall “badness of fit” of the model, with non-significant *p*-values indicating that the predictions by the model did not significantly deviate from actual data and accordingly that the fit is acceptable. The CFI assesses the relative fit of the model and compares it to a baseline model of independence (i.e., when correlations or covariances are zero). The GFI assesses the fit between the hypothesized model and the observed covariance matrix. The AGFI accounts for the complexity of the model by adjusting the GFI value according to the ratio of the number of degrees of freedom used in the model relative to the total number of degrees of freedom available. A value equal or higher than .95 indicates a good fit for the CFI, GFI and AGFI indices (Browne & Cudeck, 1992; Hu & Bentler, 1999). The RMSEA attempts to compensate for the tendency of the *χ*^2^ test to reject models with large samples or large numbers of variables. RMSEA values less than .08 indicate an acceptable fit, less than .05 a good fit, and less than .01 an excellent fit (Browne & Cudeck, 1992). The SRMR is also a “badness of fit” index as it measures the square root of the averaged squared residuals (i.e., the discrepancy between the observed and the model-implied covariances in standardized form). SRMR values of less .08 indicate good model fit (Hu & Bentler, 1999). Finally, we also report Akaike Information Criterion values (AIC; Akaike, 1973) and Bayesian Information Criterion values (BIC; Schwarz, 1978) to compare between different models. The AIC balances model fit with model complexity, lower AIC indicates better relative model fit. The BIC penalizes model complexity and thus favors parsimonious models, smaller BIC values indicate a better model fit. In addition to the fit indices, we also evaluated the models based on the direction and significance of the standardized factor loadings as Nye (2023) emphasizes that relying solely on fit indices can obscure areas of misfit. These indices, while informative, are limited in their ability to detect problems within specific parts of the model. Therefore, fit indices should not be considered sufficient on their own for selecting the optimal model.

## Results

### Descriptive Statistics

For a full overview of the descriptive statistics for the NASA Task Load Index per task, the Reaction Time task, the demographic questionnaire and the RSPM, we refer to Supplementary Table 4. Table 4 below shows participants’ performance on each cognitive control task based on the cleaned data (i.e., after removal of participant and trial outliers, transformations and removal of multivariate outliers).

**Table 4:** Descriptive Statistics (Mean Error Rates and Median Reaction Times) for the Outcome Measures of the Nine Cognitive Control Tasks.


TASK	DEPENDENT MEASURE	*MEAN (SD)*	RANGE

Antisaccade	Mean ERR	0.04 (0.05)	[0.00;0.30]

Go No-Go	Mean ERR NoGo trials (commission errors)	0.11 (0.08)	[0.00;0.45]

Stroop	Median RT Congruency Effect	95.93 (99.40)	[–99.12;408.06]

Local-Global	Median RT Switch Cost	349.00 (195.77)	[–117.11;904.12]

Number-Letter	Median RT Switch Cost	148.51 (114.89)	[–14.22;623.48]

Plus-Minus	Time Switch Cost	14.03 (17.55)	[–35.91;77.22]

Keep Track	Mean ERR	0.38 (0.10)	[0.11;0.67]

Letter Memory	Mean ERR	0.12 (0.06)	[0.01;0.30]

N-back	Mean ERR Hit trials (omission errors)	0.09 (0.08)	[0.00;0.43]


*Note*. The error-rate based measures were arcsine transformed. The Stroop task and Number-Letter task were square root and cube root transformed, respectively. For interpretability, all values presented in this table were back-transformed to the original scale. All RT-based measures are shown in milliseconds, with the exception of the Plus-Minus task, which is shown in seconds. Error-rates are shown as proportions. Means, standard deviations, and ranges were calculated based on individual participants’ mean error rates or median reaction times.

### Correlations Between the Nine Cognitive Control Tasks

To get a first indication of whether the tasks measure the same or different cognitive control functions, Pearson correlations were calculated (see Table 5). In general, the magnitude of the correlations was small (Cohen, 1988). The tasks within the updating factor were significantly, albeit weakly to moderately correlated with each other, except for the correlation between the N-back and the Letter-Memory tasks. The same was observed for the shifting factor where the Plus-Minus and the Local-Global tasks were not significantly correlated with each other. Within the inhibition factor, there was only a significant correlation between the Antisaccade and the Go No-Go tasks.

**Table 5:** Pearson Correlation Matrix between the Nine Cognitive Control Tasks.


TASK	ANTISACCADE (I)	GO NO-GO (I)	STROOP (I)	LOCAL-GLOBAL (S)	PLUS-MINUS (S)	NUMBER-LETTER (S)	KEEP TRACK (U)	LETTER MEMORY (U)	N-BACK (U)

Antisaccade (I)	–								

Go No-Go (I)	.13*	–							

Stroop (I)	.10	.07	–						

Local-Global (S)	.07	.01	–.07	–					

Plus-Minus (S)	.10	.10	.01	.11					

Number-Letter (S)	.08	.03	.17**	.18**	.15*	–			

Keep Track (U)	.11*	.11	.12	–.03	.15*	.15*	–		

Letter Memory (U)	.10	.17**	.12	.07	.20**	.07	.29***	–	

N-back (U)	.22***	.06	.13*	.05	.01	.23***	.20**	.12	–


*Note*. I = Inhibition, S = Shifting, U = Updating. * *p* < .05, ** *p* < .01, *** *p* < .001.

### Confirmatory Factor Analyses

Using CFA, we fitted the one-factor model with a common cognitive control function on which all nine tasks load, the three-factor model with three tasks each loading on the three separate cognitive control functions and where we assume that the three latent factors are correlated with each other, and finally the bi-factor model with a common cognitive control factor and two nested factors on which the updating and shifting tasks load. Fit statistics of the fitted models are presented in Table 6. The results are discussed separately by model below.

**Table 6:** Fit Indices of Fitted Latent Variable Models.


MODEL	ONE-FACTOR	THREE-FACTOR	Bi-FACTOR	Bi-FACTOR WITH VARIANCE CONSTRAINT

*χ* ^2^	40.08 (*p* = .050)	34.65 (*p* = .074)	27.25 (*p* = .163)	27.34 (*p* = .198)

CFI	.85	.88	.93	.94

GFI	.97	.97	.98	.98

AGFI	.94	.94	.95	.95

RMSEA	.04	.04	.04	.03

SRMR	.05	.05	.04	.04

AIC	5650.95	5651.52	5650.12	5648.22

BIC	5714.12	5725.21	5734.94	5728.93


*Note*. CFI = Comparative Fit Index; GFI = Goodness of Fit Index; AGFI = Adjusted Goodness of Fit Index; RMSEA = Root Mean Square Error of Approximation; SRMR = Standardized Root Mean Square Residual; AIC = Akaike Information Criterion; BIC = Bayesian Information Criterion.

#### One-Factor Model

**Figure 3:**
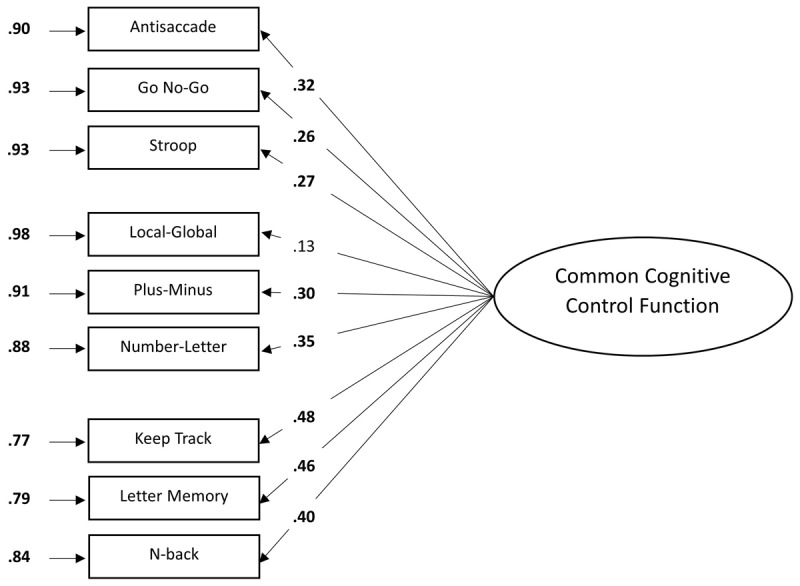
Factor loadings of the one-factor model. *Note*. The standardized factor loadings are shown on the longer arrows. The residual variances of each task due to measurement error and idiosyncratic task requirements are shown adjacent to the arrows next to each task. For all parameters, boldface type indicates *p* < .05. The latent variable is depicted as an ellipse and the rectangles represent the dependent measures of the cognitive control tasks that load on this latent variable.

Figure 3 shows the results of the one-factor model. All standardized factor loadings of the one-factor model were significant (*p* < .05) except the Local-Global task (*p* = .138) and ranged from low to moderate (.13 to .48). The tasks measuring updating showed generally higher loadings on the common cognitive control function compared to the tasks measuring inhibition and shifting. Error variances were relatively high. These high error variances indicated that a large proportion of the variance in the measures could not be accounted for by a latent common cognitive control function. All loadings were positive, in line with our expectations. The fit of the model was acceptable *χ*^2^ (27, *N* = 247) = 40.08, *p* = .050, CFI = .85, GFI = .97, AGFI = .93, RMSEA = .04, SRMR = .05, AIC = 5650.95, BIC = 5714.12. Neither the CFI nor the AGFI reached the cut-off level and the *χ*^2^ test was also not significant. According to the CFI, RMSEA and the SRMR indices, the one-factor model provided a good fit to the data. Taken together, the one-factor model, assuming unity of cognitive control, seemed to be a modest fit to the data.

#### Three-Factor Model

**Figure 4:**
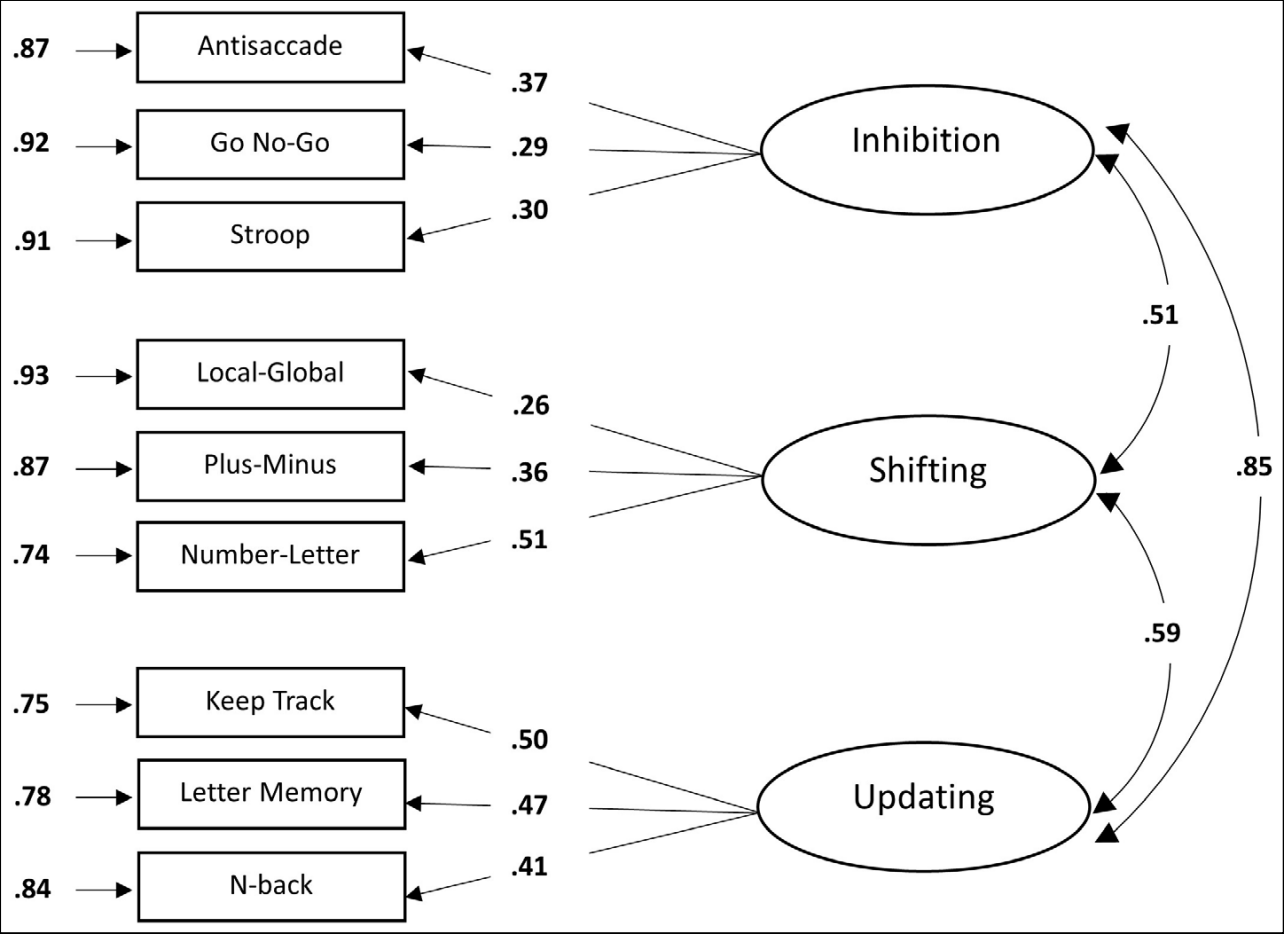
Factor loadings of the full three-factor model. *Note*. The standardized factor loadings are shown on the straight, single-headed arrows. The residual variances of each task due to measurement error and idiosyncratic task requirements are shown adjacent to the single-headed arrows next to each task. The double-headed arrows indicate the correlations between the latent variables. For all parameters, boldface type indicates *p* < .05. Latent variables are depicted as ellipses and the rectangles represent the dependent measures of the cognitive control tasks that load on these latent variables.

Figure 4 shows the results of the three-factor model. Compared to the one-factor model, all standardized factor loadings of the three-factor model were significant (*p* < .05) and ranged from low to high (.26 to .51). The factor loadings of the updating factor appeared to be higher than those of the shifting and inhibition factors, with the factor loadings of the inhibition factor showing the lowest factor loadings. Again, error variances were relatively high, suggesting that a large proportion of the inhibition, shifting and updating measures could not be accounted for by an inhibition, shifting or updating factor. All loadings were positive, in line with our expectations. Correlations between the latent variables were relatively strong (ranging from .51 to .85). The three-factor model provided a relatively good fit to the data, *χ*^2^(24, *N* = 247) = 34.65, *p* = .074, CFI = .88, GFI = .97, AGFI = .94, RMSEA = .04, SRMR = .05, AIC = 5651.52, BIC = 5725.21. Again, both the CFI and the AGFI did not reach the cut-off level. The *χ*^2^ test was non-significant in this model. According to the *χ*^2^ test, the CFI, RMSEA and the SRMR indices, the three-factor model provided a good fit to the data. From these results, we can conclude that the three-factor model, assuming both unity and diversity between the three cognitive control functions, provided a very good fit to the data.

#### Bi-Factor Model

**Figure 5:**
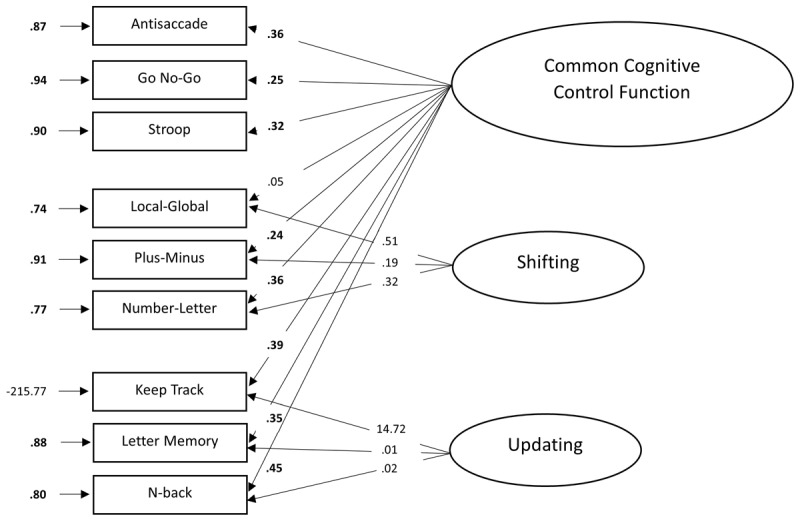
Factor loadings of the bi-factor model. *Note*. The standardized factor loadings are shown on the longer arrows. The residual variances of each task due to measurement error and idiosyncratic task requirements are shown adjacent to the arrows next to each task. For all parameters, boldface type indicates *p* < .05. Latent variables are depicted as ellipses and the rectangles represent the dependent measures of the cognitive control tasks that load on these latent variables.

Figure 5 shows the results of the bi-factor model. The fit of the bi-factor model appears to be good, *χ*^2^(21, *N* = 247) = 27.25, *p* = .163, CFI = .93, GFI = .98, AGFI = .95, RMSEA = .04, SRMR = .04, AIC = 5650.12, BIC = 5734.34. All fit indices, except for the CFI (CFI = .93) reached their cut-off levels. Similar to the results of the one-factor model, all tasks except for the Local-Global task loaded significantly on the common cognitive control factor. The loadings on the common factor ranged from low to moderate (.05 to .45) and, as expected, were all positive. The error variances were again relatively high. When looking at the factor loadings of the shifting and updating tasks on the shifting- and updating-specific factors, some issues occurred. None of these tasks loaded significantly on the shifting- and updating-specific factors. The results for the updating factor were particularly unexpected, as one item showed a factor loading greater than one (14.72), which is impossible in the population. Moreover, the model was not identified, as indicated by a non-positive definite variance-covariance matrix and negative estimated variances.

A standardized factor loading greater than one is referred to as a “Heywood case” (Harman & Fukuda, 1966). When the standardized factor loading exceeds one, it will produce a negative variance (–215.77 in our model), which is an instance of an improper solution because a negative variance is impossible in the population (Bollen, 1987). A review by Farooq (2022) showed several potential causes of Heywood cases, including small sample sizes, model misspecifications, missing data, an insufficient number of items per construct, and the possibility that the data originate from a non-interpretable model. The same review outlines various methods to address Heywood cases, with fixing negative variances to zero being the most commonly used solution (Dillon et al., 1987; Janis et al., 2016; Nachtigall et al., 2003). However, while constraining the variance to a plausible value can render the factor solution admissible, it may still compromise the validity of the construct. Therefore, such adjustments should be applied with caution, and the results interpreted critically within the broader context of the model.

**Figure 6:**
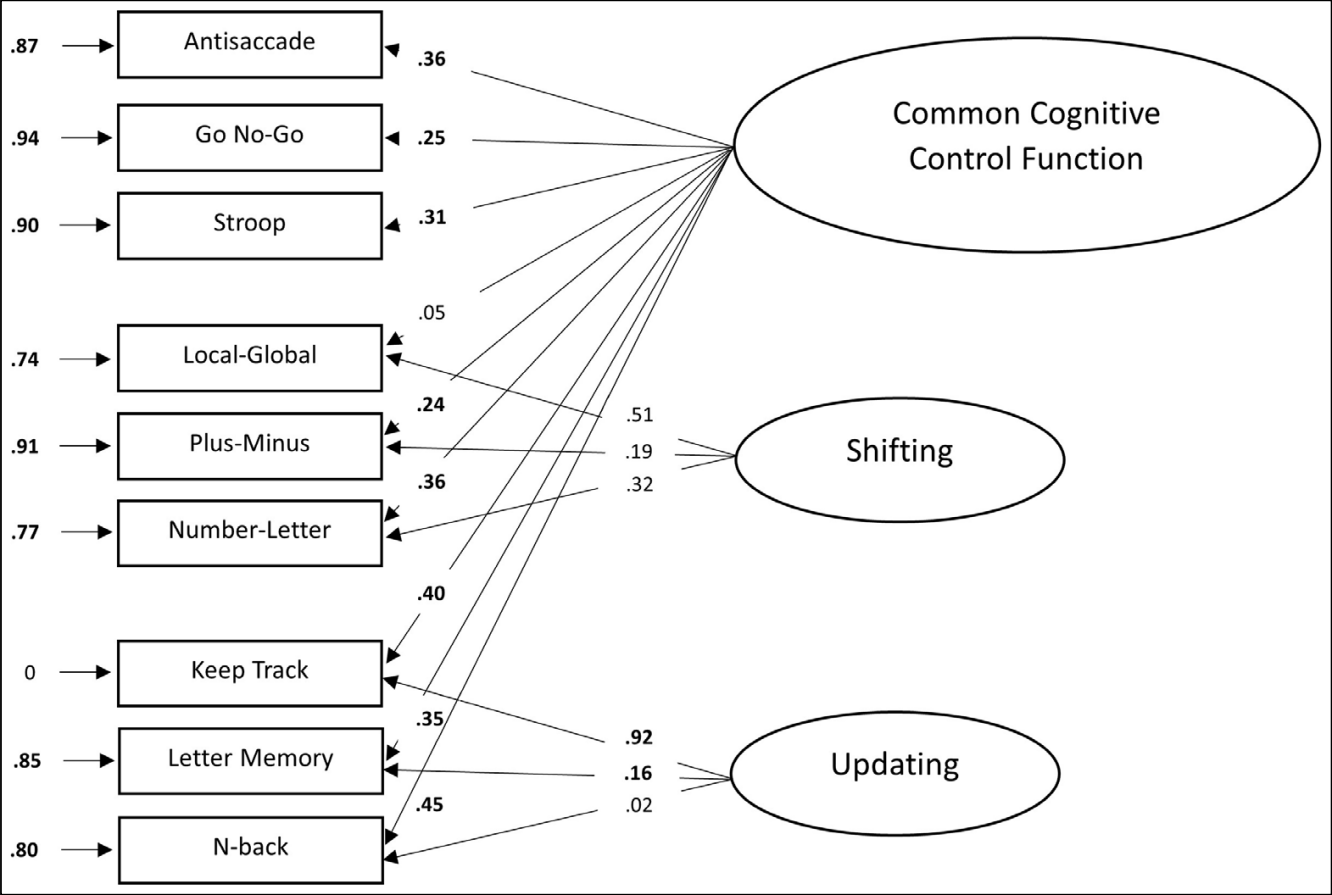
Factor loadings of the bi-factor model with the variance of the Letter-Memory task set to zero. *Note*. The standardized factor loadings are shown on the longer arrows. The residual variances of each task due to measurement error and idiosyncratic task requirements are shown adjacent to the arrows next to each task. For all parameters, boldface type indicates p < .05. Latent variables are depicted as ellipses and the rectangles represent the dependent measures of the cognitive control tasks that load on these latent variables.

Figure 6 shows the results of the bi-factor model with the variance of the Keep Track task set to zero. Similar to the previous bi-factor model, all standardized factor loadings, except for the Local-Global task, showed significance on the common cognitive control factor, with loadings nearly identical to those in the initial fit of the bi-factor model. The factor loadings on the shifting- and updating-specific factors also closely matched the fit of the initial bi-factor model, except for the Keep Track (.92), which reflects the variance constraint set to zero and the Letter Memory task, which now showed a significant factor loading (.16). The error variances of the other tasks remained almost entirely unchanged. The fit of this bi-factor model was very good, *χ*^2^(22, *N* = 247) = 27.34, *p* = .198, CFI = .94, GFI = .98, AGFI = .95, RMSEA = .03, SRMR = .04, AIC = 5648.22, BIC = 5728.93. Again, all fit indices, except for the CFI (CFI = .94) reached their cut-off levels, however fixing a negative variance to zero is known to reduce the fit indices (Farooq, 2022). Although the bi-factor model demonstrated a slightly better fit according to most fit indices, the BIC penalized this model for its complexity. In addition, several factor loadings were not significant and some estimation issues arose. Therefore, we do not consider this model, which assumes unity between the three cognitive control functions and diversity between two nested shifting and updating functions to be the optimal model.

#### Model Comparison

To compare the three fitted models, we compared the standardized factor loadings and the fit indices between the three fitted models. First, based on the standardized factor loadings, we saw that the three-factor model provided the greatest number of significant loadings and overall higher loading values compared to the other models. All but one of the loadings were also significant in the one-factor model, although these loadings were not as strong as in the three-factor model. The bi-factor model showed non-significant factor loadings on the updating- and shifting-specific factors. Second, based on the fit indices, we observed a slight improvement in model fit as complexity increased, with the bi-factor model (i.e., the most complex model) providing the best fit. This pattern is expected rather than coincidental, as more complex models often capture data structure more accurately and thus tend to show better absolute fit. Consistent with this, the AIC favored the bi-factor model (Akaike, 1973), which balances fit and complexity more leniently. In contrast, the BIC favored the more parsimonious one-factor model, penalizing the complexity of both the three-factor model and the bi-factor model more strongly (Schwarz, 1978).

## Discussion

Cognitive control is omnipresent in daily life. The unity and diversity framework of Miyake et al. (2000) distinguished inhibition, shifting and updating as important cognitive control functions. Their three-factor model set a precedent for a myriad of studies investigating the factor structure of these cognitive control functions in different populations. However, results on the factor structure are still mixed. Thus, our goal was to validate the factor structure of a new cognitive control test battery based on often-used tasks that assesses inhibition, updating and shifting. To do so, we evaluated and compared various, well-established models. Moreover, the aim of the present study was not to merely offer a replication of what has been done before, but rather to do so in full transparency and openness, reporting in detail about each step in the analysis process, openly sharing our data and tasks, and integrating the study within the current ongoing debates in the field. This open science approach could lead to scrutiny and replication efforts from others, and ultimately to theoretical and methodological advances.

To this end, data were analyzed from 247 young adults, who completed nine cognitive control tasks. Using CFA, we fitted three models: (1) a one-factor model, (2) a three-factor model; and (3) a bi-factor model. Based on the results, we contend that the three-factor model provides the best fit relative to the one-factor and bi-factor models. The three-factor model yielded the highest factor loadings, which were all positive and significant, unlike the other models, which included at least one non-significant loading. Remarkably, most factor loadings on the shifting- and updating-specific factors in the bi-factor model were not significant. However, selecting a single best-fitting model involves a trade-off, as some fit indices pointed to different conclusions. For example, the GFI, RMSEA and SRMR fit indices met their respective cut-off values in all models, while the CFI threshold was not reached in any model. Information criteria further highlighted this trade-off: the AIC and AGFI favored the bi-factor model, whereas the BIC supported the more parsimonious one-factor model. Among the three models, the one-factor model is the most parsimonious, and the bi-factor model is the most complex. Therefore, information criteria that do not heavily penalize model complexity, such as the AIC (Akaike, 1973), favored the bi-factor model, whereas those that do apply stronger penalties, such as the BIC (Schwarz, 1978), preferred the more parsimonious one-factor model. Crucially, the three-factor model was not selected as the best-fitting model by these information criteria, likely because it is neither the most parsimonious nor the most comprehensive in terms of modeled variance. While the bi-factor model showed the best overall fit based on most fit indices (except the BIC), its interpretability is limited due to non-significant factor loadings. Given these issues, caution is warranted in identifying the bi-factor model as the best-fitting model.

As mentioned earlier, the initial bi-factor model was not identified. This was indicated by the factor loading of the Keep Track task exceeding one (14.72), a Heywood case (Harman & Fukuda, 1966). This resulted in an impossible negative variance (–215.77), signaling an improper solution (Bollen, 1987). Heywood cases can arise from factors such as model misspecifications, poorly defined constructs and small sample sizes (Farooq, 2022). According to one rule of thumb for Structural Equation Modeling (Siddiqui, 2013; Stevens, 2009), 15 observations are required for each measured variable. As we included nine tasks in our CFA models, we would need at least 9 times 15 or 135 participants in total, which was achieved with our final sample size of 247 participants. This was in line with sample sizes in similar studies (cf. Karr et al., 2018, for an overview), where sample sizes were often even smaller (e.g., N = 137 in the original paper of Miyake et al., 2000). Based on this rule of thumb, our sample size thus appears sufficient and unlikely to be the cause of this Heywood case. However, other CFA guidelines recommend even larger samples than the one in the present study (Comrey & Lee, 1992; MacCallum et al., 1999; Mundfrom et al., 2005; Wolf et al., 2013). A simulation study of Kretzschmar and Gignac (2019) already showed that larger samples than the one in the present study might be needed to obtain sufficient power in studies with low reliability, which is often the case in the field of cognitive control. This study is already a step forward, as it increased the sample size compared to earlier work in this field. Even so, future research should aim for larger samples to strengthen conclusions and obtain robust results.

In summary, while the bi-factor model showed a slightly better fit on most fit indices, the BIC penalized this model for its complexity. Moreover, issues with non-significant factor loadings and estimation arose. These issues prevent us from identifying it as the optimal model. Because all factor loadings in the three-factor model were significant and were higher than in the other two models, and the model fit was adequate, we contend that the three-factor model provides the best relative fit. Nevertheless, selecting a single best-fitting model ultimately depends on the criteria emphasized: while some fit indices favored the bi-factor model (i.e., the AGFI and AIC), a case could also be made for the one-factor model according to the BIC. Furthermore, our data show that our test battery can indeed be used to assess inhibition, shifting and updating as separate but related cognitive control functions.

### A Closer Look at the Three-factor Model

Looking at this three-factor model in more detail, some results stand out. First of all, this model shows strong correlations between the three factors in the model, ranging from .51 to .85. This is not uncommon in models of cognitive control. In line with Miyake et al. (2000) and Friedman et al. (2016), our findings replicate the consistent observation that updating and inhibition are more strongly related than other pairs of cognitive control functions. As Miyake et al. (2000) discussed, successful performance on tasks requiring updating often involves inhibiting outdated or irrelevant information, and conversely, inhibition tasks (especially those involving conflict resolution) may rely on updating or working memory resources (Nyberg et al., 2009). Second, we note that in Miyake et al. (2000), the second-best fitting model was the one that combined inhibition and updating into a single factor. This indicates that at least part of the variance in the updating and inhibition tasks can be explained by a common underlying factor that captures these shared processes. In aging research, such integration appears to be even more pronounced. For example, in Adrover-Roig et al. (2012), updating and inhibition were modeled as a single factor in older adults. Likewise, Karr et al. (2018) concluded that most studies in aging populations support a two-factor solution. Third, the high correlations between latent variables may also be partially attributed to methodological constraints. Following Asparouhov and Muthén (2009) and Marsh et al. (2014), it has been shown that CFA models with overly restrictive zero cross-loadings tend to produce inflated factor correlations and distort the true latent structure. This occurs because the imposed simple structure forces the model to attribute all shared variance between tasks to the correlations among factors, rather than allowing for more nuanced item-level cross-loadings. In sum, the observed high correlations between latent variables and particularly between inhibition and updating, likely reflect both a genuine functional overlap between these cognitive control processes and the methodological constraints of the CFA framework, which may artificially inflate factor correlations due to its reliance on a simplified measurement structure.

Overall, weak correlations (ranging from .01 to .29) were observed between the nine tasks in our test battery, which is in line with findings of previous studies (Friedman & Miyake, 2004; Gärtner & Strobel, 2021; Miyake et al., 2000). The variability in the magnitude of the correlations in our study is also reflected in the reliabilities we observed of the dependent measures in the tasks in our study (see Table 3; Hedge et al., 2018; Rey-Mermet et al., 2018; Stahl et al., 2014). Significant weak correlations were observed between tasks within the same function. This indicates that the tasks used to assess the same cognitive control function partially tapped into that underlying function, indicating unity. Overall, these correlations within tasks assessing the same functions (especially the updating tasks) were weak but greater than correlations between tasks measuring different functions, indicating diversity.

However, significant correlations were also observed between tasks from different cognitive control functions. For example, the Stroop task (indexing inhibition) correlated with the Number-Letter task (indexing shifting), the Antisaccade task (indexing inhibition) with the N-back task (indexing updating), and the Number-Letter task (indexing shifting) with the N-back (indexing updating). This is in line with previous studies that also found significant correlations between tasks from different cognitive control functions (e.g., Friedman et al., 2016). These correlations could indicate that these tasks may (at least partially) tap into a more general underlying cognitive control function, again indicating a certain level of unity between the tasks. In short, the correlational data suggested that the three cognitive control functions show signs of both unity and diversity.

In addition, the strength of the factor loadings of the tasks on their corresponding underlying factor differed between factors in the three-factor model. The inhibition factor exhibited the lowest factor loadings (ranging from .29 to .37), while the updating factor demonstrated the highest factor loadings (ranging from .41 to .50). Similarly, the correlations between the inhibition tasks were generally lower and fewer of the correlations were significant compared to the correlations between the shifting tasks and the updating tasks. Furthermore, the correlations between the updating tasks were generally higher compared to the correlations between the shifting tasks. This pattern of factor loadings and correlations corroborates previous findings (e.g., Redick et al., 2016; Rey-Mermet et al., 2019; Sambol et al., 2023). They show support for the presence of a solid updating factor, while the inhibition factor has mostly been shown to be the weakest factor in young adult samples (Friedman et al., 2008; Rey-Mermet et al., 2019). The low factor loadings as well as the weak correlations between the inhibition tasks observed in our study call the presence of a common underlying inhibition factor into question (Rey-Mermet et al., 2018). When measuring inhibition, it might be better to distinguish multiple subcomponents of inhibition. Some studies already provide support for different inhibition functions (Pettigrew & Martin, 2014; Rey-Mermet et al., 2018; Stahl et al., 2014). Future research testing models with multiple inhibition factors indexed by sufficient tasks measuring each of these factors could aid in a further finetuning of the underlying factor structure of these cognitive control functions.

### Current Debates and Recommendations for Future Research

From our data, it becomes clear that cognitive control tasks are often not pure measures of the factor they are presumed to load on. This becomes evident from the high correlations between factors and the significant correlations between tasks that load onto different factors that are still present in the models. This indicates some commonality between cognitive control factors, but alternatively also highlights measurement impurity of the tasks. For example, as Miyake et al. (2000) discussed, successful performance on tasks requiring updating often involves inhibiting outdated or irrelevant information, and conversely, inhibition tasks (especially those involving conflict resolution) may rely on updating or working memory resources (Nyberg et al., 2009). Likewise, Frischkorn et al. (2022) found that updating tasks often also require more basic working memory maintenance ability, next to updating per se. In a similar vein, the Antisaccade task has been shown to involve multiple subprocesses next to inhibition, including processing speed (Rouder et al., 2022) and procedural binding ability (Frischkorn & Oberauer, 2025). Future studies could aim to isolate specific subprocesses within tasks more. Still, despite the likely involvement of multiple subprocesses in each task, CFA allows us to separate task-specific variance from shared variance (Gallagher & Brown, 2013).

In addition, as mentioned above, it may be better to include several subcomponents of inhibition into the model. Specifically, response inhibition and interference control have been identified as two separable inhibitory control functions, although different frameworks may use slightly different terminology (Bunge et al., 2002; Friedman & Miyake, 2004; Noreen & MacLeod, 2015; Rey-Mermet et al., 2018). Response inhibition refers to the ability to suppress or inhibit responses that are prepotent or automatic in nature, whereas interference control refers to the ability to remain focused on the task at hand and suppress or inhibit irrelevant or disruptive information due to resource or stimulus competition (Friedman & Miyake, 2004). Indeed, emerging literature is increasingly showing that these two subcomponents of inhibition can clearly be distinguished, both behaviorally (Friedman & Miyake, 2004; Rey-Mermet et al., 2018) and at the neural level (Long et al., 2022). The Antisaccade task and the Go No-Go task are thought to primarily measure response inhibition, whereas the Stroop task is thought to primarily measure interference control (Gärtner & Strobel, 2021; Rey-Mermet et al., 2018). Although small, our data revealed a significant correlation (.13) between the two presumed response inhibition tasks (i.e., the Antisaccade and the Go No-Go tasks). No significant correlations were observed between the response inhibition tasks and the interference control task (i.e., the Stroop task). These findings suggest that a model with separate response inhibition and interference control factors may provide an even better fit to the data. However, testing such a model would require multiple tasks for both inhibition factors.

The low and non-significant correlations between tasks of a same latent factor not only support the distinction between inhibition subcomponents, but also reflect a broader issue in cognitive psychology: the often surprisingly low correlations between tasks that are assumed to tap into the same construct. The low correlations observed in this study align with previous findings highlighting weak associations between cognitive control tasks (Friedman & Miyake, 2004; Frischkorn et al., 2019; Gärtner & Strobel, 2021; von Bastian et al., 2020). These low correlations often reflect unaccounted trial-level noise (Mehrvarz & Rouder, 2025) and the limited reliability of the tasks and their outcome measures. A central issue is that robust experimental effects, such as the Stroop congruency effect, are often ill-suited for individual differences research (Hedge et al., 2018; von Bastian et al., 2020). Experimental tasks typically maximize within-subject and condition-level variance, but restrict between-subject variance, limiting their usefulness for correlational approaches (Hedge et al., 2018; Rouder et al., 2023; Snijder et al., 2024; Zorowitz & Niv, 2023). Consequently, while these tasks yield reliable group effects, they often fail to capture meaningful interindividual differences. Brysbaert (2024) offers practical guidelines for designing and evaluating experimental tasks and for combining them to more effectively study individual differences in cognitive control.

In addition, difference scores seem to be especially problematic, as subtracting conditions (e.g., incongruent and congruent conditions) decreases the already limited variability even more (Burgoyne et al., 2023; Draheim et al., 2019; Vandierendonck, 2017; von Bastian et al., 2020). Thus, the implications of this low reliability mainly depend on how the tasks are used in studies rather than on their validity. Although increasing the number of trials may help (Hedge et al., 2018; Lee et al., 2025), it does not always solve the issue and is not always practical in large test batteries or studies involving clinical or developmental populations (von Bastian et al., 2020). To address high trial-level noise, researchers have also proposed more advanced analytic approaches. For example, Holmén et al. (2024) applied a network analysis, selected for its independence from latent variables, and compared it to factor analysis, but concluded that the network approach likewise failed to reveal convincing higher-order groupings of cognitive control function measures. Another promising strategy is the use of hierarchical models, which can separate trial-level noise from true individual variability. Freund et al. (2025) reported that this approach yielded more precise correlation estimates and allowed reliable individual differences to be detected. However, other authors were less optimistic, noting that even when measurement error was reduced through hierarchical modeling, correlations remained imprecise and no higher-order cognitive control factor emerged (Rey-Mermet et al., 2025; Rouder et al., 2023). They therefore argued that the difficulty in establishing cognitive control as a psychometric construct reflects more than just a measurement problem.

Another recommendation we would like to make for future research is to assess and validate this cognitive control test battery in more heterogeneous younger adult populations with different levels of socio-economic status (i.e., education and income), a broader age range and a more balanced biological sex and gender distribution. Moreover, our sampling method resulted in a WEIRD sample, as we recruited undergraduate students who participated for course credit. Consequently, the sample was relatively homogeneous in age (17 to 24 years), biological sex (84.21% of whom were female), and racial/ethnic background. In particular, the strong sex imbalance prevented us from testing the robustness of the models across sex, for example, using multisample analysis (Byrne, 2004). Future studies should include a larger number of male participants to enable this robustness check. Therefore, caution is warranted when generalizing our findings to the broader young adult population. Notably, the open-source nature of our test battery allows researchers to assess and validate the battery in more heterogeneous young adult samples.

Moreover, it is recommended to increase uniformity in this field. Previous literature often showed different factor structures in similar samples (e.g., Garcia-Barrera, 2019; Karr et al., 2018), or failed to report well converging models (e.g., Karr et al., 2018; Sambol et al., 2023). Given the wide variety of different cognitive control tasks that have been used, which are often not openly and/or freely available, we believe that a uniform, easily accessible and adaptable open-source cognitive control test battery will aid the field. The current study therefore developed a cognitive control test battery, with a well-known underlying factor structure. This test battery, available both in Dutch as well as in English, is openly available on OSF. The selected tasks are suitable for use in different populations, and can be used as a test battery, but each task can also be used in a stand-alone manner. We encourage other researchers to start using these tasks, enhancing uniformity in the field, as well as to further adapt and enhance this cognitive control test battery, under the principles of open science.

Some additional recommendations remain when adopting this test battery. We deliberately selected cognitive control tasks that are commonly used in the literature and adaptable to a wide range of populations. In particular, we focused on tasks that do not require vocal responses, allowing for group administration and making the battery suitable for developmental and aging populations, such as children and older adults. The selected tasks are similar to those often used in these age groups (Hull et al., 2008; Otsuka et al., 2021; Schnitzspahn et al., 2012; Soto et al., 2021; Vaughan & Giovanello, 2010). Future studies can tailor the tasks based on the specific needs of their target population, for example, by adjusting response deadlines, adjusting stimulus complexity, inserting more or fewer breaks between task blocks, providing more extensive instructions, changing the number of practice trials, or shortening the test sessions. Notably, we have already started using these tasks in older adults (De Pue et al., 2025). The open-source nature of the battery supports such adaptations and promotes its validation and consistent use in diverse developmental populations. The factor structure of cognitive control, and more specifically the presence of inhibition, shifting and updating factors, remains unclear in children and older adults. While some studies replicated the three-factor structure of Miyake et al. (2000) (Fisk & Sharp, 2004; Lehto et al., 2003; Vaughan & Giovanello, 2010), others suggested a dedifferentiation of cognitive control functions in early and later stages of the lifespan, providing evidence for models with only one or two underlying factors (Adrover-Roig et al., 2012; Karr et al., 2018; Lee et al., 2013; van der Ven et al., 2013). However, these studies, especially in older adults, have often used complex tasks or neuropsychological tests instead of tasks targeting specific cognitive control functions. Assessment of the test battery discussed in this study in different age groups could enhance further exploration of the development of the factor structure underlying these cognitive control tasks.

Second, task selection is an important consideration: other choices would have been valid, and task choice can have an impact on correlations between tasks and the obtained factor structure (Friedman, 2016; Karr et al., 2018; Sambol et al., 2023; von Bastian et al., 2020). Third, different studies exploring the factor structure of cognitive control functions have used different ways of cleaning the data. Moreover, not all studies reported the cut-offs they used to exclude outlying trials in such detail that this could be replicated by other researchers. In fact, this study is one of the few to transparently report data cleaning and analysis procedures when examining the factor structure of common cognitive control tasks. Similarly, in this literature, if and how assumptions were checked and how violations of assumptions were handled is often not clear. For example, whereas most studies use the arcsine transformation to achieve normality for error-rate based measures in line with Miyake et al. (2000), non-normality and corresponding transformations for reaction times are often not discussed. This field would benefit from a more transparent and detailed description of data cleaning procedures. We therefore openly share the code of the current study, available on OSF via https://osf.io/vtpjb/, so others can replicate or use our step-by-step cleaning and analysis procedure. Furthermore, we encourage researchers to adapt the tasks and explore the dataset, as we see open science as key to improving the psychometric validity of cognitive control measures (von Bastian et al., 2020).

## Conclusion

To summarize, to address the mixed findings in the literature on the factor structure of cognitive control tasks, we fitted a one-factor, a three-factor and a bi-factor model in young adults using a cognitive control test battery consisting of nine tasks designed to assess inhibition, shifting and updating. Our findings were most consistent with a three-factor structure in which inhibition, shifting and updating emerged as separate but correlated factors. However, only some fit indices provided mixed evidence and pointed toward the bi-factor model and the BIC suggested a possible one-factor solution. This study is among the few that transparently details data cleaning and analysis procedures when examining the factor structure of commonly used tasks. The full test battery is openly shared, providing the field with a uniform set of freely available tasks with an established factor structure that can be applied across diverse populations to study cognitive control. We encourage researchers to adapt the test battery and explore the dataset further. We believe this openscience approach is essential for addressing challenges in the field and advancing toward more psychometrically valid measures of cognitive control (von Bastian et al., 2020).

## Data Accessibility Statement

The anonymized, raw and aggregated data that support the findings of this study, the test battery used in this study, and the R code used to generate the results are available in the Open Science Framework (OSF, https://osf.io/vtpjb/).

## Additional File

The additional file for this article can be found as follows:

10.5334/joc.480.s1 Supplementary Information. Supplementary Material 1, Supplementary Tables 1–7 and Supplementary Figures 1–3.
