# Supplementary material for: Miyake Revisited: Validating the Factor Structure of an Open-Source Cognitive Control Test Battery
Source: J Cogn. 2026 Jan 8;9(1):8. doi: 10.5334/joc.480 (PMC12785704; doi:10.5334/joc.480)
Supplement: Supplementary Information. — Supplementary Material 1, Supplementary Tables 1–7 and Supplementary Figures 1–3. [file joc-9-1-480-s1.pdf]

Miyake revisited: Validating the factor structure of an open-source cognitive control test battery

-

Supplementary Information

Supplementary Information

Supplementary Material 1 – Cognitive control tasks 03

Supplementary Table 1 – Different task orders 09

Supplementary Table 2 - Spearman correlation matrix between the nine cognitive control tasks based on the uncleaned dataset.. 10

Supplementary Table 3 - Pearson correlation matrix between the nine cognitive control tasks based on the uncleaned dataset.. 11

Supplementary Table 4 - Fit indices of fitted latent variable models based on the uncleaned dataset 12

Supplementary Table 5 – Summary of univariate statistics after several transformations for enhancing data normality. 13

Supplementary Table 6 – Summary of univariate statistics after each data cleaning step 15

Supplementary Table 7 - Descriptive Statistics for the NASA Task Load Index, reaction time task, demographic questionnaire and Raven SPM 17

Supplementary Figure 1 - Factor loadings of the one-factor model based on the uncleaned dataset 18

Supplementary Figure 2 - Factor loadings of the full three-factor model based on the uncleaned dataset 19

Supplementary Figure 3 - Factor loadings of the bi-factor model based on the uncleaned dataset ... 20

Supplementary Material 1

The **Antisaccade task** (Roberts et al., 1994) measures inhibition of a dominant tendency to make a saccade toward a cue rather than a saccade away from the cue to identify the target. A fixation cross was presented for a jittered duration between 1500-3500 ms in 250 ms intervals. Subsequently, a cue (i.e., a small square, width = 0.4, height = 0.4) appeared on one side of the screen for 225 ms, followed by a target on the opposite side of the screen for 25 ms, after which this target was masked by a black and white grid (width = 2, height = 2). The target stimulus was an arrow pointing either up, left or right (width = 2, height = 2). To be able to perform this task well, participants must look in the opposite direction of the flashed cue, inhibiting the reflexive tendency to saccade to the cue. Participants had to indicate the direction of the arrow with a left, right or up key press response as quickly and accurately as possible, within 2500 ms after the target presentation. After an inter-trial interval (ITI) of 2000 ms, the next trial started. After 12 practice trials with feedback, participants received 90 main trials in two blocks, with a self-paced pause between each block. There were 30 trials each for the target with the arrow pointing left, right and up. These 90 trials were presented in random order. The proportion of errors was used as the main dependent measure, indexing *inhibition*.

The **Go No-Go task** (adapted from Logan et al., 1984) measures response inhibition. A continuous stream of fixation crosses was presented for 500 ms alternated with colored squares (blue or yellow; width = 4, height = 4) also presented for 500 ms. Participants were instructed to press the "J" key as quickly and accurately as possible when the square was blue (i.e., Go trial) and to withhold a key press when the target was yellow (i.e., No-Go trial). This rule was counterbalanced across participants. Go trials were more frequent (i.e., 80% of the trials) than No-Go trials, so that the Go trials became the prepotent response. Participants completed 20 practice trials with feedback and 200 main trials in four blocks, with a self-paced pause between each block. Feedback was also provided after each incorrect main trial. The dependent measure in this task was the commission error rate (i.e.,

percentage of errors on No-Go trials, when the participant pressed a button when a response should have been inhibited), indexing *inhibition*.

The **Stroop task** (adapted from Stroop, 1935) measures inhibition of the automatic response of reading a color word while naming the ink color of the color word. First, a fixation cross was presented for 1000 ms, followed by the color word (size = 1.125) until the participant responded. These color words were either printed in the same ink color as the meaning of the word (congruent trial) or in a different color (incongruent trial). Participants were instructed to respond to the color of the stimuli as quickly and accurately as possible. On incongruent trials, participants had to actively inhibit reading the word to be able to respond to the ink color. Four different colors were used, and participants used one response key for each color ("D" = red, "F" = green, "J" = blue, "K" = yellow). Participants completed 15 practice trials with feedback, followed by 48 main trials (24 congruent and incongruent trials each). The dependent measure was the congruency effect, which refers to the interference caused by the automatic reading of the word. The congruency effect was calculated as the difference between the median RT of incongruent trials and the median RT of congruent trials, indexing *inhibition*.

The **Local-Global task** (Navon, 1977) measures the ability to switch between identifying the global and local features of a target stimulus. On each trial, participants were presented with a large object composed of smaller objects (e.g., a triangle composed of small circles). The shapes could be either circles, squares, crosses or triangles. First, a fixation cross was presented on the screen for 500 ms. Then the target (width = 10, height = 10) was presented and participants were instructed to respond as quickly and accurately as possible to either the global feature (the large object) or the local feature (the smaller objects that make up the large object) while ignoring the uninstructed feature. A blue or yellow frame (width = 11, height = 11, line width = 8) around the target indicated respectively a global or local task rule. This rule was counterbalanced across participants. Participants had to respond within 20 s, using the keys "D", "F", "J" and "K" corresponding to the possible shapes of the

object. The response mapping was counterbalanced and was also projected on the bottom of the screen at all times. Importantly, on half of the trials, participants had to switch from examining the local features to the global features or vice versa from one trial to the next trial. Participants received 24 practice trials with feedback and 96 main trials, divided in two blocks of 48 trials with a self-paced pause. The dependent measure was the switch cost, which was calculated as the difference between the median RTs of the trials that required a shift in the mental set (i.e., color of the frame changed) and the trials that required no shift (i.e., the color of the frame remained the same), indexing *shifting*.

The **Plus-Minus task** (adapted from Jersild, 1927 and Spector & Biederman, 1976) measures task switching between mathematical operations. Participants were presented with three blocks of two-digit random numbers (size = 40). For the first block, participants had to add 3 to each two-digit number, for the second block they had to subtract 3 from each number, and for the final block they had to switch between adding 3 and subtracting 3. Each block was presented on a single screen, where participants completed a list of 30 numbers, presented in three columns of 10 numbers. Participants had to type their answer after each number and then continue to the next number on the list as quickly and accurately as possible. Before each main block, participants were first given a practice block of 6 numbers and received feedback on their performance. The dependent measure was the switch cost, which was calculated as the difference between the time required to complete the alternating block and the average of the time required to complete the addition and subtraction blocks, indexing *shifting*.

The **Number-Letter task** (adapted from Rogers and Monsell, 1995) measures the ability to switch rapidly between a number and letter classification task. On each trial of this task, a number-letter pair (e.g., “7G”, size = 1) was presented in the middle of the computer screen. A cue (i.e., “even or odd?” or “vowel or consonant?”, size = 1) asked participants to either classify the number (i.e., number classification task) or classify the letter (i.e., letter classification task) as quickly and accurately as possible. Cues were presented for 1000 ms. The first two blocks consisted solely of pure number or

letter classification tasks, with the order of the blocks being randomized. The final block was a mixed block where participants had to switch between the two classification tasks from trial to trial. The “F” and “J” keys were used to respond and response mapping was counterbalanced. Participants received 12 practice trials with feedback followed by three main blocks of 64 (number classification), 64 (letter classification) and 128 trials (both number and letter classification), with a self-paced pause between each block. The dependent measure was the switch cost, calculated as the difference between the median RTs for the switch trials in the mixed block and the average of the median RTs of the trials in pure number and letter classification blocks, indexing *shifting*.

The **Keep Track task** (Yntema, 1963) measures the ability to update working memory representations. At the start of the task, participants were presented with a set of categories and a list of possible words per category on the screen (size = 1). Categories included: animals, colors, countries, relatives, metals and clothes. At the beginning of each trial, participants were first shown the categories that would be of interest in that trial for 3000 ms in the center of the screen. Next, these target categories remained at the bottom of the screen, and words from these categories were serially and randomly presented for 1500 ms each (size = 1), with three words being presented for each category. Participants had to remember the last word presented for each of the target categories, and were asked to type in the last word of each target category at the end of the trial. For example, if one of the target categories was “color” and participants saw “yellow”, “aluminum”, “pants”, …, “blue”, they were supposed to type “blue” at the end of the trial. Participants received one practice trial with three target categories. The main task consisted of three trials with four target categories and three trials with five target categories, with a total of 27 stimuli to recall. The dependent measure was the proportion of errors (i.e., the proportion of words that the participant identified incorrectly), indexing *updating*.

The **Letter Memory task** (adapted from Morris & Jones, 1990) required participants to update their working memory representation continuously. During each trial, a random series of 5, 7, 9 or 11

letters (size = 0.9) was presented serially on the screen. A fixation cross was first presented for 1000 ms. This was followed by presentation of the letter string, with each letter being presented for 2000 ms. The participants' task was to recall the last four letters that were presented in the list. For example, if the letters presented were ''T, H, G, B, S, K, R,'' the participants should remember, ''T . . . TH . . . THG . . . THGB . . . HGBS . . . GBSK . . . BSKR'' and then recalled ''BSKR'' at the end of the trial. Participants responded by typing in the letters with the keyboard at the end of the trial. The order of the letters was not important. Participants received a practice block of four trials with 5 or 7 letters with feedback, followed by a main block of 25 trials with random letter streams with 4, 5, 7, 9 or 11 letters. The trials with four letters were removed from analysis as there was no updating involved on these trials. The dependent measure was the proportion of errors (i.e., the proportion of letters that were incorrectly recalled), indexing *updating*.

The **N-back task** (adapted from Kirchner, 1958) also measures the ability to update working memory representations. Grayscale images of objects and animals (width = 281/45, height = 197/45) were presented serially for 1500 ms. All the images used were validated in a previous study by Rossion and Pourtois (2004). On each trial, participants had to decide as quickly and accurately as possible whether the presented image was identical to the image that was presented two trials back (i.e., 2-back task), by pressing the "J" key if this was the case (i.e., a hit trial). The interstimulus-interval was 500 ms. Participants received one practice block of 50 trials with feedback and two main blocks of 50 trials, both with a 30% hit trials. A self-paced pause was provided between the two main blocks. Feedback was also provided after each incorrect main trial. The dependent measure was the omission error rate (i.e., the proportion of errors on hit trials, when participants did not press a button when a button should have been pressed), indexing *updating.* Omission errors occur more frequently than commission errors in N-back tasks and are more strongly associated with working memory capacity (Meule, 2017; Oberauer, 2005).

Finally, participants also had to complete a simple **reaction time task** to measure their general processing speed. They had 1500 ms to respond to a white square (width = 4, height = 4), with intertrial intervals varying between 500 ms and 4000 ms. Participants had to respond as quickly as possible by pressing the “J” key. They received 10 practice trials and 50 main trials. Median RTs were calculated to measure general processing speed.

Supplementary Table 1

Different Task Orders

Task order	Order A	Order B	Order C	Order D	Order E	Order F
Task 1	Antisaccade	Antisaccade	Plus-Minus	Plus-Minus	Number-Letter	Number-Letter
Task 2	Local-Global	Local-Global	Letter Memory	Letter Memory	N-back	N-back
Task 3	Keep Track	Keep Track	Go No-Go	Go No-Go	Stroop	Stroop
Task 4	Plus-Minus	Number-Letter	Antisaccade	Number-Letter	Antisaccade	Plus-Minus
Task 5	Letter Memory	N-back	Local-Global	N-back	Local-Global	Letter Memory
Task 6	Go No-Go	Stroop	Keep Track	Stroop	Keep-Track	Go No-Go
Task 7	Number-Letter	Plus-Minus	Number-Letter	Antisaccade	Plus-Minus	Antisaccade
Task 8	N-back	Letter Memory	N-back	Local-Global	Letter Memory	Local-Global
Task 9	Stroop	Go No-Go	Stroop	Keep Track	Go No-Go	Keep Track

Supplementary Table 2

Spearman Correlation Matrix between the Nine Cognitive Control Tasks Based on the Uncleaned Dataset

Task	Antisaccade (I)	Go No-Go (I)	Stroop (I)	Local-Global (S)	Plus-Minus (S)	Number-Letter (S)	Keep Track (U)	Letter Memory (U)	N-back (U)
Antisaccade (I)	-								
Go No-Go (I)	.13*	-							
Stroop (I)	.04	.07	-						
Local-Global (S)	.10	.03	-.04	-					
Plus-Minus (S)	.14*	.15*	0.00	.09	-				
Number-Letter (S)	.09	.00	.17**	.18**	.22***	-			
Keep Track (U)	.12*	.20***	.12*	-.03	.13*	.09	-		
Letter Memory (U)	.18**	.19**	.05	.00	.20***	.06	.30***	-	
N-back (U)	.24***	.11	.10	.07	.08	.27***	.21***	.22***	-

Note. I = Inhibition, S = Shifting, U = Updating. * $p < .05$, ** $p < .01$, *** $p < .001$. Correlations that are significant in the uncleaned data, but were not significant in the cleaned data are highlighted in green. Correlations that are not significant in the uncleaned data, but were significant in the cleaned data are highlighted in yellow.

Supplementary Table 3

Pearson Correlation Matrix between the Nine Cognitive Control Tasks Based on the Uncleaned Dataset

Task	Antisaccade (I)	Go No-Go (I)	Stroop (I)	Local-Global (S)	Plus-Minus (S)	Number-Letter (S)	Keep Track (U)	Letter Memory (U)	N-back (U)
Antisaccade (I)	-								
Go No-Go (I)	.20**	-							
Stroop (I)	.00	.05	-						
Local-Global (S)	.01	.05	-.01	-					
Plus-Minus (S)	.17**	.11	-.01	.07	-				
Number-Letter (S)	-.01	.02	.13*	.13*	.24***	-			
Keep Track (U)	.22***	.22***	.12*	-.01	.13*	.08	-		
Letter Memory (U)	.32***	.24***	.08	.04	.16**	-.01	.36***	-	
N-back (U)	.21***	.13*	.03	.10	.09	.24***	.26***	.30***	-

Note. I = Inhibition, S = Shifting, U = Updating. * *p* < .05, ** *p* < .01, *** *p* < .001. Correlations that are significant in the uncleaned data, but were not significant in the cleaned data are highlighted in green. Correlations that are not significant in the uncleaned data, but were significant in the cleaned data are highlighted in yellow.

Supplementary Table 4

Fit Indices of Fitted Latent Variable Models Based on the Uncleaned Dataset

Model	One-factor	Three-factor	Bi-factor
χ^2	53.83 (p = .002)	38.26 (p = .033)	35.19 (p = .027)
CFI	.85	.92	.92
GFI	.96	.97	.97
AGFI	.94	.95	.94
RMSEA	.06	.05	.05
SRMR	.06	.05	.05
AIC	10787.13	10777.57	10780.49
BIC	10852.81	10854.19	10868.07

Note. CFI = Comparative Fit Index; GFI = Goodness of Fit Index; AGFI = Adjusted Goodness of Fit Index; RMSEA = Root Mean Square Error of Approximation; SRMR = Standardized Root Mean Square Residual; AIC = Akaike Information Criterion ; BIC = Bayesian Information Criterion.

Supplementary Table 5

Summary of Univariate Statistics After Several Transformations for Enhancing Data Normality

Task	None	Log	Square	Square root	Cube root	Inverse	Yeo-Johnson	Arcsine	Logit
**Antisaccade**									
Skewness	2.10	1.90	4.63	0.47	-0.39	-1.72	2.32	0.57	0.67
Kurtosis	5.31	4.17	26.02	0.18	-0.10	3.23	6.69	0.39	-0.02
Shapiro-Wilk	0.77	.80	.44	.95	.93	.82	.75	.95	.94
	($p < .001$)	($p < .001$)	($p < .001$)	($p < .001$)	($p < .001$)	($p < .001$)	($p < .001$)	($p < .001$)	($p < .001$)
**Go No-Go**									
Skewness	1.30	1.08	3.04	0.07	-0.85	-0.87	1.55	0.24	0.01
Kurtosis	1.77	0.99	11.61	0.17	1.69	0.40	2.80	0.29	-0.19
Shapiro-Wilk	0.89	.91	.63	.98	.93	.93	.86	.97	.98
	($p < .001$)	($p < .001$)	($p < .001$)	($p < .001$)	($p < .001$)	($p < .001$)	($p < .001$)	($p < .001$)	($p < .001$)
**Stroop**									
Skewness	0.80	-6.69	1.90	-0.24	-1.19	-1.75	3.25		
Kurtosis	1.18	78.92	5.04	3.32	9.59	62.98	14.05		
Shapiro-Wilk	0.96	.62	.84	.96	.92	.24	.61		
	($p < .001$)	($p < .001$)	($p < .001$)	($p <.001$)	($p < .001$)	($p < .001$)	($p < .001$)		
**Local-Global**									
Skewness	0.34	-9.91	1.88	-0.94	-2.14	-4.65	3.28		
Kurtosis	1.65	117.66	7.31	5.39	15.36	51.03	16.56		
Shapiro-Wilk	0.98	.49	.87	.94	.87	.40	.71		
	($p < .001$)	($p < .001$)	($p < .001$)	($p < .001$)	($p < .001$)	($p < .001$)	($p < .001$)		
**Plus-Minus**									
Skewness	0.42	-6.91	1.67	-0.84	-1.89	-0.65	3.91		
Kurtosis	1.43	80.45	5.05	5.76	14.12	49.68	20.98		
Shapiro-Wilk	0.98	.61	.89	.94	.89	.43	.59		
	($p < .001$)	($p < .001$)	($p < .001$)	($p < .001$)	($p < .001$)	($p < .001$)	($p < .001$)		

**Number-Letter**									
Skewness	1.26	-1.50	2.62	0.37	-0.03	3.41	2.69		
Kurtosis	1.39	5.69	7.64	-0.08	0.08	21.08	8.06		
Shapiro-Wilk	0.90	.91	.66	.99	.99	.50	.65		
	($p < .001$)	($p < .001$)	($p < .001$)	($p = .013$)	($p = .427$)	($p < .001$)	($p < .001$)		
**Keep Track**									
Skewness	0.13	-0.12	1.04	-0.38	-0.56	0.38	0.39	-0.06	-0.30
Kurtosis	0.26	0.21	1.72	0.48	0.75	0.35	0.50	0.43	0.79
Shapiro-Wilk	0.98	.98	.93	.98	.97	.98	.98	.98	.98
	($p = .004$)	($p = .004$)	($p < .001$)	($p < .001$)	($p < .001$)	($p <.001$)	($p < .001$)	($p = .002$)	($p < .001$)
**Letter Memory**									
Skewness	0.51	0.40	1.46	-0.10	-0.35	-0.29	0.62	-0.02	-0.79
Kurtosis	-0.22	-0.35	2.19	-0.27	0.05	-0.45	-0.04	-0.29	1.20
Shapiro-Wilk	0.97	.98	.86	.99	.98	.98	.96	.99	.96
	($p < .001$)	($p < .001$)	($p < .001$)	($p = .077$)	($p = .005$)	($p = .003$)	($p < .001$)	($p = .106$)	($p <. 001$)
**N-back**									
Skewness	1.39	1.10	4.16	-0.17	-0.85	-0.86	1.72	-0.03	-0.12
Kurtosis	2.78	1.54	23.09	-0.34	-0.08	0.67	4.54	-0.17	-0.56
Shapiro-Wilk	0.88	.90	.55	.94	.87	.92	.85	.94	.95
	($p < .001$)	($p < .001$)	($p < .001$)	($p < .001$)	($p < .001$)	($p < .001$)	($p < .001$)	($p < .001$)	($p < .001$)

Supplementary Table 6

Summary of Univariate Statistics After Each Data Cleaning Step

Task	Untrimmed	Participant outliers removed	+ Trial outliers removed	+ Transformations executed	+ Multivariate outliers removed
**Antisaccade**					
Skewness	3.75	2.10		0.57	0.47
Kurtosis	16.38	5.31		0.39	0.30
Shapiro-Wilk	0.56	0.77		0.95	0.95
	($p < .001$)	($p < .001$)		($p < .001$)	($p < .001$)
**Go No-Go**					
Skewness	1.25	1.30		0.24	0.14
Kurtosis	1.50	1.77		0.29	0.23
Shapiro-Wilk	0.89	0.89		0.97	0.97
	($p < .001$)	($p < .001$)		($p < .001$)	($p < .001$)
**Stroop**					
Skewness	1.27	0.77	0.80	-0.24	0.37
Kurtosis	3.76	1.66	1.18	3.32	-0.01
Shapiro-Wilk	0.92	0.96	0.96	0.96	0.99
	($p < .001$)	($p < .001$)	($p < .001$)	($p < .001$)	($p = .014$)
**Local-Global**					
Skewness	0.10	0.24	0.34		-0.08
Kurtosis	1.22	1.31	1.65		-0.30
Shapiro-Wilk	0.99	0.98	0.98		0.99
	($p = .008$)	($p = .006$)	($p < .001$)		($p = .521$)
**Plus-Minus**					
Skewness	0.38	0.42			0.23
Kurtosis	2.13	1.43			0.14
Shapiro-Wilk	0.96	0.98			0.99
	($p < .001$)	($p < .001$)			($p = .191$)

**Number-Letter**					
Skewness	1.38	1.40	1.26	-0.03	-0.07
Kurtosis	1.94	2.05	1.39	0.08	0.23
Shapiro-Wilk	0.88	0.88	0.90	0.99	> 0.99
	(p < .001)	(p < .001)	(p < .001)	(p = .427)	(p = .646)
**Keep Track**					
Skewness	0.71	0.13		-0.06	-0.29
Kurtosis	2.59	0.26		0.43	0.29
Shapiro-Wilk	0.96	0.98		0.98	0.98
	(p < .001)	(p = .004)		(p = .002)	(p = .001)
**Letter Memory**					
Skewness	1.54	0.51		-0.02	-0.04
Kurtosis	6.25	-0.22		-0.29	-0.25
Shapiro-Wilk	0.91	0.97		0.99	0.99
	(p < .001)	(p < .001)		(p = .106)	(p = .164)
**N-back**					
Skewness	1.37	1.39		-0.03	-0.12
Kurtosis	2.43	2.78		-0.17	-0.38
Shapiro-Wilk	0.88	0.88		0.94	0.94
	(p < .001)	(p < .001)		(p < .001)	(p < .001)

Note. No trial outliers were removed for ERR measures, as these measures were binary scored. The Local-Global and Plus-Minus tasks were not transformed in the end.

Supplementary Table 7

Descriptive Statistics for the NASA-task Load Index, Reaction Time Task, Demographic Questionnaire and Raven SPM

Measure	Mean (*SD*)	
**NASA Task Load Index**	Subjective Performance Index	Subjective Mental Effort Index
Antisaccade	31.03 (21.17)	56.62 (25.17)
Go No-Go	24.09 (17.63)	45.91 (23.56)
Stroop	18.06 (14.63)	40.51 (21.65)
Local-Global	44.62 (23.02)	73.16 (17.48)
Plus-Minus	13.68 (16.65)	49.82 (23.97)
Number-Letter	33.08 (16.97)	63.00 (18.95)
Keep Track	75.57 (17.73)	79.09 (16.11)
Letter Memory	43.72 (19.01)	71.38 (17.92)
N-back	30.77 (19.38)	57.89 (21.44)
**Reaction Time task**		
Median RT	255 (24)	
**Demographic questionnaire**		
Normal numbers of hours of sleep	7.79 (0.88)	
Number of hours of sleep last night	7.27 (1.25)	
Normal number of cups of coffee per day	0.50 (0.81)	
Number of cups of coffee in the past 24 hours	0.43 (0.89)	
Normal number of glasses of alcohol per week	3.16 (4.72)	
Number of glasses of alcohol in the past 24 hours	0.28 (1.37)	
**Demographic questionnaire**	*N*	
Color blindness	0	
Diseases with known influence on cognitive functioning	10	
Medication with known influence on cognitive functioning	6	
**Raven Standard Progressive Matrices**	Raw score	Percentile score
	50.83 (4.81)	54.45 (25.04)

Note. RTs are shown in milliseconds. Both subscales of the NASA-task Load Index were scored on a scale of 0 to 100 in increments of five, where 0 corresponded to ‘perfect’ and 100 to ‘failure’ for the Subjective Performance Index. For the Subjective Mental Effort Index, 0 corresponded to ‘very low’ and 100 to ‘very high’.

Supplementary Figure 1

Factor Loadings of the One-Factor Model Based on the Uncleaned Dataset

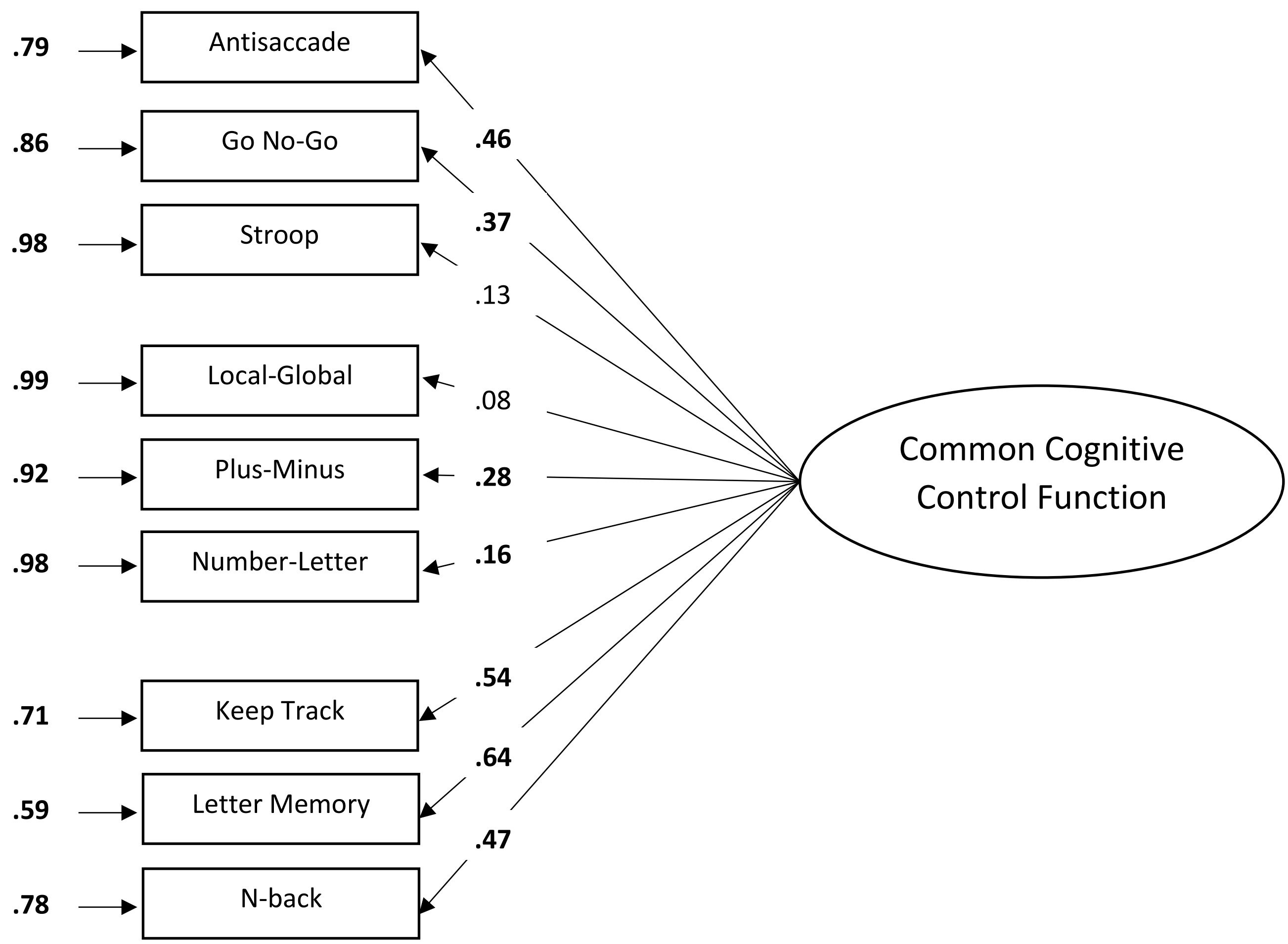

Note. The standardized factor loadings are shown on the longer arrows. The residual variances of each task due to measurement error and idiosyncratic task requirements are shown adjacent to the arrows next to each task. For all parameters, boldface type indicates $p < .05$. The latent variables is depicted as an ellipse and the rectangles represent the dependent measures of the cognitive control tasks that load on this latent variable.

Supplementary Figure 2

Factor Loadings of the Full Three-Factor Model Based on the Uncleaned Dataset

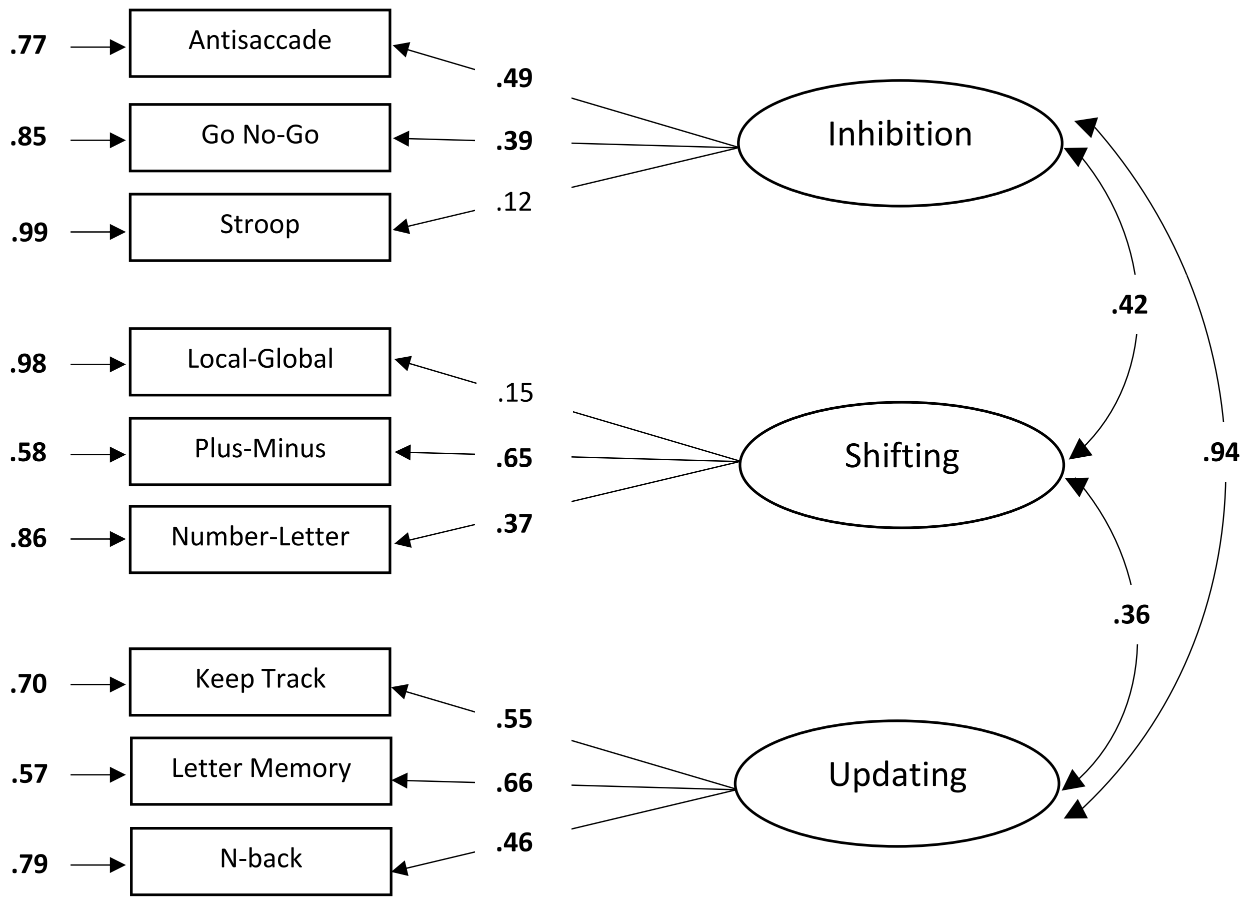

Note. The standardized factor loadings are shown on the straight, single-headed arrows. The residual variances of each task due to measurement error and idiosyncratic task requirements are shown adjacent to the single-headed arrows next to each task. The double-headed arrows indicate the correlations between the latent variables. For all parameters, boldface type indicates $p < .05$. Latent variables are depicted as ellipses and the rectangles represent the dependent measures of the cognitive control tasks that load on these latent variables.

Supplementary Figure 3

Factor Loadings of the Bi-Factor Model Based on the Uncleaned Dataset

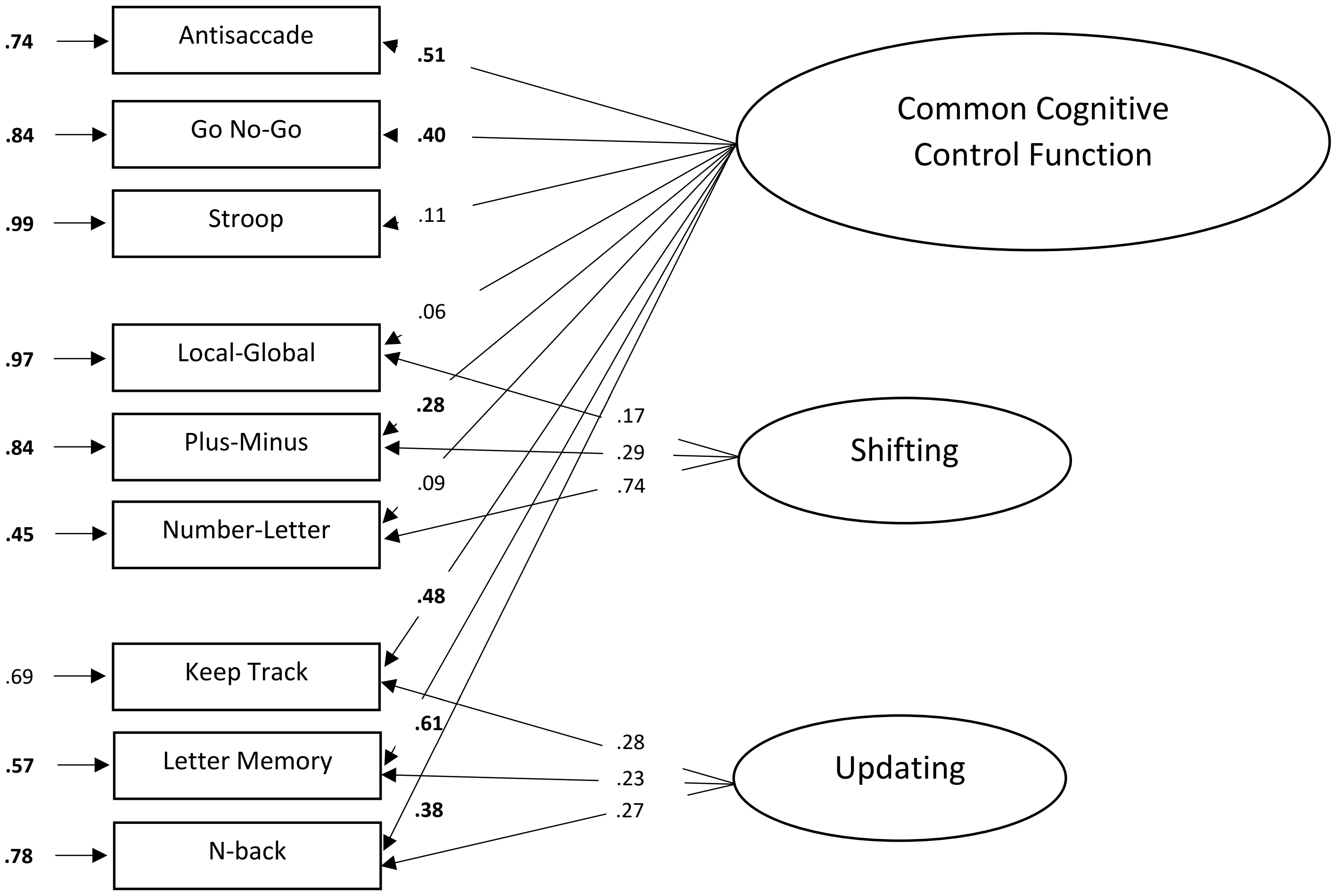

Note. The standardized factor loadings are shown on the longer arrows. The residual variances of each task due to measurement error and idiosyncratic task requirements are shown adjacent to the arrows next to each task. For all parameters, boldface type indicates $p < .05$. Latent variables are depicted as ellipses and the rectangles represent the dependent measures of the cognitive control tasks that load on these latent variables.

References

Jersild, A. T. (1927). Mental set and shift. *Archives of Psychology*, *89*

Kirchner, W. K. (1958). Age differences in short-term retention of rapidly changing information. *Journal of Experimental Psychology*, *55*(4), 352–358. https://doi.org/10.1037/h0043688

Logan, G. D., Cowan, W. B., & Davis, K. A. (1984). On the ability to inhibit simple and choice reaction time responses: A model and a method. *Journal of Experimental Psychology. Human Perception and Performance*, *10*(2), 276–291. https://doi.org/10.1037//0096-1523.10.2.276

Meule, A. (2017). Reporting and interpreting working memory performance in n-back tasks. *Frontiers in Psychology*, *8*, 352. https://doi.org/10.3389/fpsyg.2017.00352

Morris, N., & Jones, D. M. (1990). Memory updating in working memory: The role of the central executive. *British Journal of Psychology*, *81*(2), 111–121. https://doi.org/10.1111/j.2044-8295.1990.tb02349.x

Navon, D. (1977). Forest before trees: The precedence of global features in visual perception. *Cognitive Psychology*, *9*(3), 353–383. https://doi.org/10.1016/0010-0285(77)90012-3

Oberauer, K. (2005). Binding and inhibition in working memory: Individual and age differences in short-term recognition. *Journal of Experimental Psychology. General*, *134*(3), 368–387. https://doi.org/10.1037/0096-3445.134.3.368

Roberts, R. J., Hager, L. D., & Heron, C. (1994). Prefrontal cognitive processes: Working memory and inhibition in the antisaccade task. *Journal of Experimental Psychology: General*, *123*(4), 374–393. https://doi.org/10.1037/0096-3445.123.4.374

Rogers, R. D., & Monsell, S. (1995). Costs of a predictible switch between simple cognitive tasks. *Journal of Experimental Psychology: General*, *124*(2), 207–231. https://doi.org/10.1037/0096-3445.124.2.207

Rossion, B., & Pourtois, G. (2004). Revisiting Snodgrass and Vanderwart's object pictorial set: The role of surface detail in basic-level object recognition. *Perception*, *33*(2), 217–236. https://doi.org/10.1068/p5117

Spector, A., & Biederman, I. (1976). Mental set and mental shift revisited. *The American Journal of Psychology*, *89*(4), 669–679. https://doi.org/10.2307/1421465

Stroop, J. R. (1935). Studies of interference in serial verbal reactions. *Journal of Experimental Psychology*, *18*, 643–662. https://doi.org/10.1037/h0054651

Yntema, D. B. (1963). Keeping track of several things at once. *Human Factors*, *5*(1), 7–17. https://doi.org/10.1177/001872086300500102
